# Mitochondrial transfer from adipose-derived regenerative cells contributes therapeutic angiogenesis in a murine hindlimb ischemia model

**DOI:** 10.1007/s10456-025-10001-z

**Published:** 2025-09-10

**Authors:** Yiyang Che, Yuuki Shimizu, Takumi Hayashi, Junya Suzuki, Zhongyue Pu, Kazuhito Tsuzuki, Shingo Narita, Yoshimitsu Yura, Rei Shibata, Toyoaki Murohara

**Affiliations:** 1 Department of Cardiology, Nagoya University Graduate School of Medicine, 65 Tsurumai, Showa-ku, Nagoya, 466-8550 Japan; 2 Department of Advanced Cardiovascular Therapeutics, Nagoya University Graduate School of Medicine, Nagoya, 466-8550 Japan

**Keywords:** Hindlimb ischemia, Adipose-derived regenerative cells, Mitochondrial transfer, Angiogenesis

## Abstract

**Objective:**

Adipose-derived regenerative cells (ADRCs) are promising cell sources for damaged tissue regeneration. The efficacy of therapeutic angiogenesis with ADRC implantation in patients with critical limb ischemia has been demonstrated in clinical studies. There are several possible mechanisms in this process such as cytokines and microRNA. Recently, cell-to-cell transfer of mitochondria gains more attention in regenerative medicine. However, the role of the mitochondrial transfer mechanism in ADRCs in the regeneration of functional tissue perfusion following ischemic injury remains unclear. In this study, we aimed to investigate whether mitochondrial transfer is a potential mechanism of therapeutic angiogenesis in ADRCs using a murine hindlimb ischemia model.

**Methods and results:**

In initial studies, the occurrence of mitochondrial transfer of ADRC to endothelial cells and macrophages in a series of pro-angiogenic effects of ADRC was demonstrated in a mouse model of hindlimb ischemia. Subsequently, we comprehensively elucidated the modes of mitochondrial transfer from ADRCs to HUVECs and macrophages mediated by Connexin43-based gap junctions and tunneling nanotubes using time-lapse confocal microscopy and cell sorting techniques. Furthermore, mitochondrial transfer from ADRCs enhanced mitochondrial biogenesis and angiogenesis in vascular endothelial cells and shifted macrophages toward the M2-phenotype. Notably, partially canceled mitochondrial transfer from ADRCs could impede the angiogenic ability of ADRCs in hind limb ischemia.

**Conclusions:**

ADRCs can protect against ischemic limbs, at least in part by mitochondrial transfer via gap junctions and tunneling of nanotubes into injured endothelial cells and macrophages. Additionally, mitochondrial transfer is a potential mechanism for therapeutic angiogenesis with ADRCs in hindlimb ischemia.

**Graphical abstract:**

Schematic illustration showing potential mechanisms of mitochondrial transfer from ADRCs in mouse hindlimb ischemia model. This figure was created with BioRender.
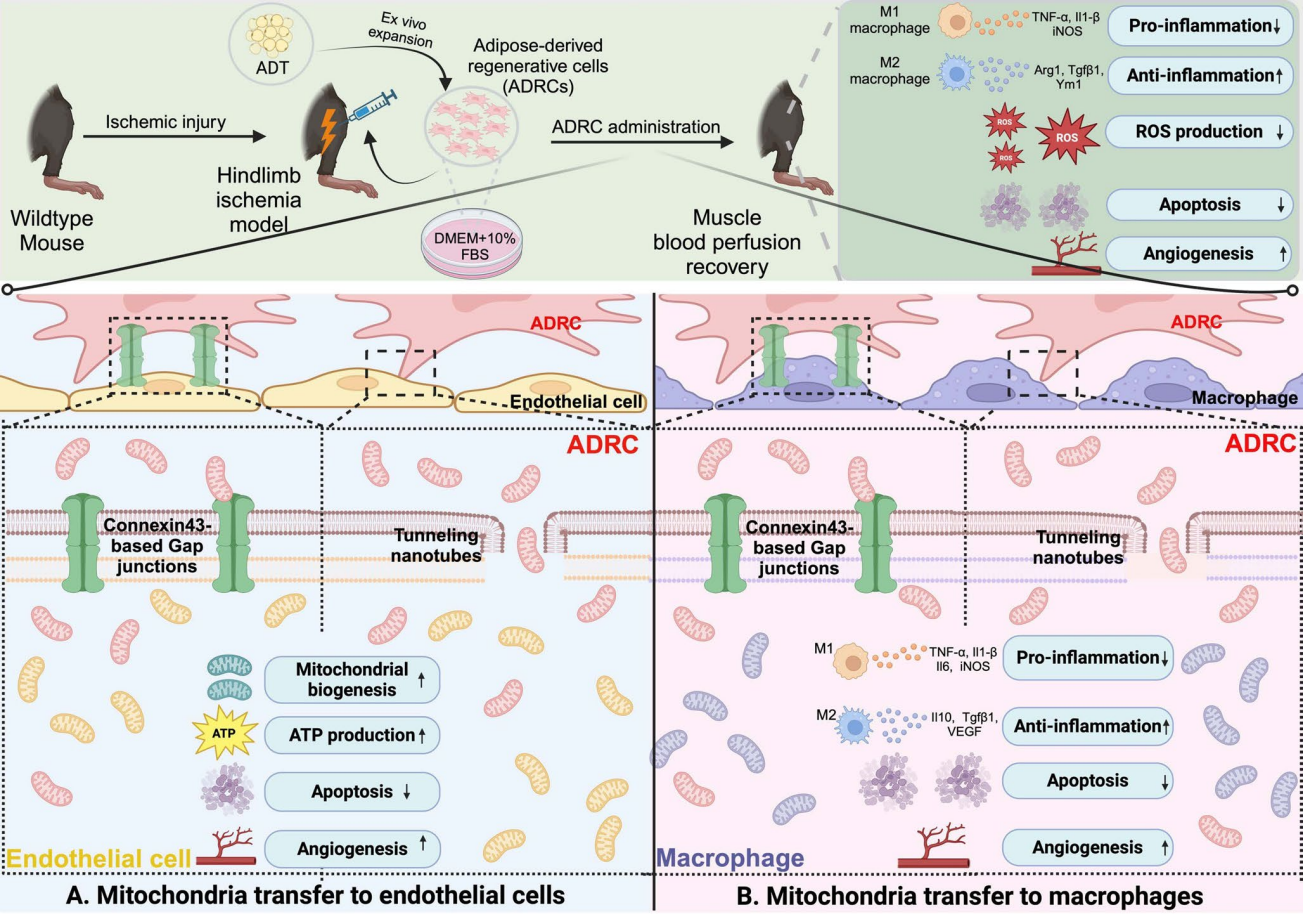

**Supplementary Information:**

The online version contains supplementary material available at 10.1007/s10456-025-10001-z.

## Introduction

Peripheral artery disease (PAD) is an obstructive atherosclerotic condition of the lower limbs that affects millions of people worldwide [1, 2]. Treatment guidelines recommend multimodal therapies, such as guidance on lifestyle habit improvement, pharmacotherapy, and revascularization. However, some patients still undergo lower-limb amputation even after comprehensive treatments. In this situation, “therapeutic angiogenesis,” is another strategy treatment for cases wherein limbs cannot be rescued by conventional treatment [3]. Subcutaneous adipose tissue contains mesenchymal stem cells (MSCs) called adipose-derived regenerative cells (ADRCs) [4]. Compared to bone marrow-derived MSCs (BM-MSCs), ADRCs are easily collected in large quantities and are less invasive; however, they have almost the same potential as bone marrow-derived cells.

In addition to the differentiation of implanted cells toward damaged target cells, paracrine effect (secretion of angiogenesis-promoting factors, such as VEGF and HGF) and/or exosome-mediated mechanism, such as microRNA, have been explored among the mechanisms underlying the protective effects of cell-based therapy [5–7]. Additionally, direct cell contacts between bone marrow-derived MSCs and endothelial cells, macrophages, or other recipient cells, which allow mitochondrial transfer, are also crucial in the protective mechanisms of MSCs. It has been demonstrated that mitochondrial transfer with direct cell interaction between MSCs and alveolar type II epithelial cells is via Connexin 43 gap junctions [8]. Moreover, in an in vitro model, mitochondrial transfer from BM-MSCs to endothelial cells via tunneling nanotube (TNT) has been shown to be protective in cultured cells [9]. MSCs could also transfer mitochondria into macrophages partially mediated through TNT-like structures [10]. Nevertheless, there a gap remains in our knowledge regarding the extent to which the therapeutic effects of ADRCs are mediated by mitochondrial transfer, as well as the specific modes of mitochondrial transfer within the context of therapeutic angiogenesis.

Accordingly, the present study aimed to investigate whether mitochondrial transfer is a potential mechanism for therapeutic angiogenesis in ADRCs and how mitochondrial transfer influences angiogenesis in a murine hindlimb ischemia model.

## Materials and methods

Materials and Methods are available in the manuscript or Supplemental File. The data, analytic methods, and study materials employed in this study are available from the corresponding author upon reasonable request.

### Animal studies

C57BL6J male mice (8–10 weeks) purchased from Charles River Laboratories Japan Inc. (Kanagawa, Japan) were used in an experiment approved by the Animal Ethics Review Board of the Nagoya University School of Medicine. Mice were randomly assigned to ischemic injury and treatment groups. Before the surgical procedure, mice were anesthetized with a combination of hydrochloric acid medetomidine (0.3 mg/kg), midazolam (4 mg/kg), and butorphanol tartrate (5 mg/kg) intraperitoneally. Cervical dislocation under anesthesia was used for euthanasia.

### Cell culture

*Isolation of mouse ADRCs*: The inguinal subcutaneous adipose tissue of mice was used for ADRCs isolation as described previously [11, 12]. The first and second passages of ADRCs were used in this study. After ADRCs reached 80% confluence, mouse Gja1 siRNA (100 nM, Dharmacon) or scrambled sequence (negative control, 100 nM, Ambion) was transfected using Lipofectamine RNAi Max (ThermoFisher Scientific) for 48 h, according to the manufacturer’s instructions. Next, the culture medium was removed, and ADRCs were treated with Latrunculin A (0.1 µM, Sigma) for 2 h and then continued to coculture experiments further [13]. The transfected and treated ADRCs were shortened as ADRC-sL in the present study.

*Isolation of mouse peritoneal macrophages (mMφ)*: Primary mouse peritoneal macrophages were isolated four days after injection of thioglycolate medium intraperitoneally as described [14] and cultured in RPMI 1640 medium (Sigma) supplemented with 10% fetal bovine serum and 1% penicillin/streptomycin overnight. Then primary mMφ was used for further coculture experiments.

Human umbilical vein endothelial cells (HUVECs) purchased from Lonza were grown in EGM-2MV (Lonza). Cells in passages 5–9 were used for this experiment.

For cell label, cells were stained with 200-500 nM MitoTracker Green FM (Invitrogen), 200–500 nM MitoTracker Red CMXRos (Invitrogen), or 500 nM CellTracker Orange CMTMR (Invitrogen) at 37 degrees for 30 min in incubator according to the manufacturer’s instructions and then washed twice followed by incubation in complete media for another 1 h before the experiments to remove the excess MitoTracker dyes [15].

For transduction with CellLight Mitochondria GFP, BacMam 2.0 (Invitrogen), 30 particles per cell were used, and incubation was continued for 20-24 h following the manufacturer’s protocol and then continued to further experiments [16].

### Mouse hindlimb ischemia model and cell transplantation

After anesthesia, hindlimb ischemia was performed by ligation of the left femoral artery and attaching branches as previously described [11, 17]. For experiments investigating ADRC-induced angiogenesis, the ADRC group was administered with 1 × 10^6^ cells resuspended in 60 µl sterile PBS into three different points of ischemic adductor muscles at ischemic day 1 per animal, and vehicle control as the PBS group received 60 µl of sterile PBS [11, 17]. To investigate mitochondrial transfer from ADRC in the hindlimb ischemia model, hindlimb ischemia mice were received 1 × 10^6^ MitoTracker Red-labeled or 1 × 10^6^ Mitochondria-GFP-labeled ADRCs per animal at ischemic day 1 and sacrificed for further immunofluorescence experiment. For other experiments studying the effect of inhibition on mitochondrial transfer from ADRCs in hindlimb ischemia, the ADRC group was injected with 1 × 10^6^ ADRCs per animal resuspended in 60 µl sterile PBS, and the ADRC-sL group was delivered with 1 × 10^6^ ADRC-sLs per animal resuspended in 60 µl sterile PBS.

### Blood flow measurements

Blood flow measurements were performed using the laser Doppler system (Moor Instruments) immediately after hindlimb ischemia surgery to evaluate the success of hindlimb ischemia model induction. Then animals were further measured for blood flow on ischemic day 7, 14, 21, and 28. The quantitative analysis of blood flow perfusion was expressed as the ratio of ischemic to non-ischemic perfusion [11, 17].

### Direct co-culture experiments

ADRC coculture experiments were established by either incubating with HUVECs in a 1:1 ratio or mMφ in a 1:10 ratio for defined time points according to different experiments. Hypoxia condition was stimulated by AnaeroPack System (MGC) in a rectangular jar with less than 0.1% of oxygen, and more than 15% of CO_2_. To induce hypoxia/reoxygenation injury, cells were further reoxygenated 24 h after being exposed with hypoxia condition 12 h (12H/24R) [18].

### Non-direct contact system with transwell inserts

ADRCs were cocultured with HUVECs in a 1:1 ratio (Figure S7A) using a 1 µm transwell insert (Corning Inc.) [19]. ADRCs were also pre-treated with 10 µM exosome inhibitor GW4869 (MCE) for 24 h to study the extracellular vesicles mode of mitochondrial transfer [20]. After the 12H/24R condition, HUVECs in the 24-well plate were collected for further experiments [19].

### Confocal microscopy and time-lapse imaging

After MitoTracker red-labeled ADRC coculture with either MitoTracker green-labeled HUVEC or MitoTracker green-labeled mMφ for 12 h of hypoxia condition, live-cell images and videos were then acquired on SpinSR10 (Evident, Tokyo, Japan) together with stage-top incubator STXF-WSKMX-SET (Tokai Hit, Shizuoka, Japan) using a 60 × or 100 × oil immersion objective. Fluorescence intensity analysis of images was assessed using Image J software [21].

### Immunofluorescence staining

Adductor muscle samples from each group at ischemic day 1, 3, and 28 were embedded in an OCT compound (Sakura). Frozen sections (8 µm in thickness) were fixated with 4% paraformaldehyde (PFA) and washed thrice with PBS, the sections were blocked with 1% BSA at room temperature for 1 h and then incubated with primary antibody anti-mouse CD31 rat mAb (1:500, BD Pharmingen), anti-mouse CD68 rat mAb (1:200, BioLegend), anti-mouse CD11c rabbit mAb (1:200, CST), or anti-mouse CD206 rat mAb (1:200, BioLegend) at 4 °C overnight, followed by incubation with secondary antibody Alexa-Fluor 594 conjugated anti-rat antibody (1:1000, Thermo Fisher Scientific), Alexa-Fluor 488 conjugated anti-rat antibody (1:1000, Thermo Fisher Scientific), or Alexa-Fluor 488 conjugated anti-rabbit antibody (1:1000, Thermo Fisher Scientific) at room temperature for 1 h. Cell nuclei were stained with 4′,6-diamidino-2-phenylindole (DAPI; 1:1000, DOJINDO). Also, frozen sections (8 µm in thickness) at ischemic day 3 were performed with dihydroethidium (DHE) staining (Invitrogen) to detect ROS generation according to the manufacturer’s instructions [22, 23]. Images were visualized using a BZ-X710 fluorescence microscope (KEYENCE, Japan) at 20 × or 60 × magnification. Five pictures were randomly taken per section, and positive cell numbers and the fluorescence intensity were analyzed using Image J software [5].

After MitoTracker green-labeled ADRC coculture with either CellTracker orange-labeled HUVEC in 12H/24R condition or CellTracker orange-labeled mMφ in 24 h of hypoxia condition, live-cell images were then taken with a BZ-X710 fluorescence microscope (KEYENCE, Japan) at 20 × magnification.

### Hematoxylin and Eosin (H&E) staining

Adductor muscle samples from each group at ischemic day 21 and 28 were embedded in an OCT compound (Sakura). Frozen sections (8 µm in thickness) were fixated with 4% paraformaldehyde (PFA) and then stained with H&E. Images were acquired using a BZ-X710 fluorescence microscope (KEYENCE, Japan) at 20× magnification. Adductor muscle H&E cross-sections were analyzed for average muscle fiber size. Five pictures were randomly taken per section, and the average muscle fiber size was assessed using Image J software [24].

#### TUNEL staining

Frozen adductor muscle sections (8 µm in thickness) at ischemic day 3 were stained with In Situ Cell Death Detection Kit, Fluorescein (Roche) and anti-mouse CD31 rat mAb (1:500, BD Pharmingen) or anti-mouse CD68 rat mAb (1:200, BioLegend) followed by incubation with secondary antibody Alexa-Fluor 594 conjugated anti-rat antibody (1:1000, Thermo Fisher Scientific) according to the manufacturer’s protocol [25]. Cell nuclei were stained with 4′,6-diamidino-2-phenylindole (DAPI; 1:1000, DOJINDO). Images were visualized using a BZ-X710 fluorescence microscope (KEYENCE, Japan) at 20 × magnification. Five pictures were randomly taken per section, and positive cell numbers were analyzed using Image J software.

### Immunocytochemistry

For evaluation of mitochondrial transfer from Mitochondria-GFP-labeled ADRCs to HUVECs or macrophages, after fixation with 4% PFA and blocking with 1% BSA, cells were incubated with anti-human CD31 rabbit mAb (1:200, Abcam) or anti-mouse CD68 rat mAb (1:200, BioLegend) at 4 °C overnight, followed by incubation with Alexa-Fluor 594 conjugated anti-rabbit antibody (1:1000, Thermo Fisher Scientific) or Alexa-Fluor 594 conjugated anti-rat antibody (1:1000, Thermo Fisher Scientific) for 1 h at room temperature according to the manufacturer’s instructions. Cell nuclei were stained with DAPI. Images were taken using a BZ-X710 fluorescence microscope (KEYENCE, Japan) at 20 × magnification.

For proliferation assay of ADRCs or BM-MSCs coculture experiments with HUVECs after 12H/24R, cells were fixated with 4% PFA and then incubated with anti-human CD31 rabbit mAb (1:200, Abcam) at 4 °C overnight, followed by incubation with Alexa-Fluor 488 conjugated Ki67 antibody (1:200, BD Pharmingen) and Alexa-Fluor 594 conjugated anti-rabbit antibody (1:1000, Thermo Fisher Scientific) for 1 h at room temperature according to the manufacturer’s instructions. Cell nuclei were stained with DAPI. Images were taken using a BZ-X710 fluorescence microscope (KEYENCE, Japan) at 20 × magnification. Five pictures were randomly taken per section, and the proliferated cell numbers were analyzed as both Ki67 positive and CD31 positive using Image J software.

For ADRCs coculture experiments with mMφs after 24 h of hypoxia, cells were fixated with 4% PFA followed by 1 h 1% BSA blocking, and then incubated with CD11c rabbit mAb (1:200, Abcam) and CD206 rat mAb (1:200, BioLegend) at 4 °C overnight, followed by incubation with Alexa-Fluor 488 conjugated anti-rabbit antibody (1:1000, Thermo Fisher Scientific) and Alexa-Fluor 594 conjugated anti-rat antibody (1:1000, Thermo Fisher Scientific) for 1 h at room temperature according to the manufacturer’s instructions. Cell nuclei were stained with DAPI. Images were taken using a BZ-X710 fluorescence microscope (KEYENCE, Japan) at 20 × magnification. Five pictures were randomly taken per section, and the cell numbers were counted either as CD11c positive or CD206 positive using Image J software.

For investigation of mitochondrial transfer mode from ADRCs to HUVECs or macrophages, after fixation with 4% PFA and blocking with 1% BSA, cells were incubated with Connexin43 rabbit mAb (1:200, CST) at 4 °C overnight, followed by incubation with Alexa-Fluor 488 conjugated anti-rabbit antibody (1:1000, Thermo Fisher Scientific) and Texas Red-X conjugated wheat germ agglutinin (5:1000, Thermo Fisher Scientific) for 1 h at room temperature according to the manufacturer’s instructions. Cell nuclei were stained with DAPI. Images were taken using a BZ-X710 fluorescence microscope (KEYENCE, Japan) at 20 × magnification.

### Migration assay

Migration assay in this study was performed with Costar transwell migration chambers (Corning Inc.). Different groups of cells after 12H/24R in EBM2 with 0.5% FBS were seeded onto the upper insert membrane (3-µm pore), then placed in a 24-well plate with EGM-2MV and incubated for 6 h. Cells were then fixated with 4% PFA for 15 min and incubated with Alexa Fluor 488 anti-CD31 antibody (1:200, Abcam) for 1 h at room temperature and followed with 4′,6-diamidino-2-phenylindole staining (DAPI; 1:1000, DOJINDO) for 3 min [5]. After thoroughly rinsed with PBS, the upper side of the membrane was gently swabbed with a cotton tip, and the migrated HUVECs were counted as CD31 positive cells using a BZ-X710 fluorescence microscope (KEYENCE, Japan) at 20 × magnification. Five pictures were randomly taken per membrane, and the migrated cell numbers were analyzed using Image J software.

### Analysis of mitochondrial mass and mitochondrial membrane potential

Mitochondrial mass was evaluated in HUVECs from coculture experiments by pre-labelling HUVECs with MitoTracker green and mitochondrial membrane potential was assessed in HUVECs from coculture experiments by pre-labelling HUVECs with MitoTracker red [26]. After 12H/24R, fluorescence intensity was acquired using plate reader SpectraMax iD5 (Molecular Devices, San Jose, CA, USA).

### ATP determination

For ATP detection of ADRCs coculture experiments with HUVECs after 12 h of hypoxia, total cellular ATP measurement was performed in cell lysates with ATP detection assay kit-luminescence (Cayman, No.700410) according to the manufacturer’s instructions.

### Electron microscopy

HUVECs or cocultured with ADRCs or ADRC-sL after 12H/24R condition were fixed with 2.5% glutaraldehyde in 0.1 M sodium phosphate buffer, pH7.4 and the post-fixed with 2% osmium tetraoxide for 1 h. Cells were then dehydrated with a series of graded ethanol and finally embedded in Epon812 resin for 48 h. Images were acquired in transmission electron microscopy JEM-1400 Flash (JEOL Ltd, Tokyo, Japan) at 100 kV [27].

### Flow cytometry

For cell sorting, a single-cell suspension was prepared from coculture experiments and then labeled with antibodies on ice for 20 min. Antibodies included PE-Cy7 conjugated anti-mouse Sca1 (BioLegend) and APC-Cy7 conjugated anti-human CD31 (BioLegend) [28]. The sorted HUVECs were collected in EGM-2MV with a 5 ml flow tube using FACS Aria Fusion (Becton, Dickinson and Company, Franklin Lakes, NJ, USA) and continued to further experiment.

### Isolation of RNA and quantitative real-time PCR analysis

RNA was extracted from adductor muscles in the hindlimb ischemia model at ischemic day 3 or cell suspensions using the RNeasy Mini Kit (Qiagen), and an equal amount of total RNA was reverse-transcribed with ReverTra Ace qPCR RT Master Mix (TOYOBO). Real-time PCR was performed using a Bio-Rad real-time PCR detection system with THUNDERBIRD SYBR qPCR mix (TOYOBO) under the following conditions: 95 °C for 10 min, followed by 40 cycles at 95 °C for 15 s and 60 °C for 45 s [29]. The gene expression was normalized to *Gapdh* or *36b4* and the primer sequences are listed in the Supplemental Materials.

### DNA extraction and mtDNA measurements

Total DNA was extracted from sorted HUVECs in coculture experiments using the DNeasy Blood & Tissue Kit (Qiagen) and an equal amount of DNA was analyzed with quantitative real-time PCR. The mtDNA expression was normalized to human Gapdh or human nuclear DNA and the primer sequences are listed in the Supplemental Materials [30].

### Western blot analysis

Adductor muscle samples from each group at ischemic day 3 or cell lysates were homogenized and prepared as previously described. Equal amounts of protein were subjected to CriterionTGX (Tris–Glycine extended) gels (BioRad) and the protein was then transferred to PVDF membranes as previously described [31]. The membranes were then blocked and probed with the targeted primary antibodies, followed by incubation with secondary antibodies. The protein signal was detected using an ECL Prime Western Blotting System (Cytiva). Protein expression was analyzed by measuring band intensities using Image J software. The antibodies used in this study are described in the Supplemental Materials.

### Seahorse analysis

Seahorse analysis was performed using a Seahorse XF24 analyzer (Agilent). Cells were seeded 10,000 per well on Seahorse XF24 microplates (Agilent) in a 12H/24R condition and were changed to XF assay medium (Agilent) in a non-CO_2_ incubator for 45–60 min before the assay.

*Mito stress test*: The mitochondrial stress test was performed using the Seahorse XF Cell Mito Stress Test Kit (Agilent), and the XF Assay Medium used in this test contained 10 mM glucose, 1 mM sodium pyruvate, and 2 mM glutamine. Activators and inhibitors were injected after each cycle and were used at the following concentrations: Oligomycin (1.5 µM), FCCP (1 μM), and Rot/AA (0.5 μM). Results were analyzed using WAVE software (Agilent) and normalized to the cell numbers following the manufacturer’s instructions [19].

*Glycolysis stress test*: The glycolysis stress test was performed using the Seahorse XF Glycolysis Stress Test Kit (Agilent), and the XF Assay Medium used in this test contained 2 mM glutamine. Activators and inhibitors were injected after each cycle and were used at the following concentrations: Glucose (10 mM), Oligomycin (1 μM), and 2-DG (50 mM). Results were analyzed using WAVE software (Agilent) and normalized to the cell numbers following the manufacturer’s instructions.

### Statistical analysis

Data were expressed as mean ± standard error of the mean (SEM) and analysis was performed with GraphPad Prism 10 software. The data normal distribution was assessed using Shapiro–Wilk’s test. For normal distribution data, statistical significance was evaluated using an unpaired Student’s *t*-test between the two groups, and a one-way analysis of variance (ANOVA) with Tukey’s multiple comparison test for three or more groups. Also, two-way repeated-measures ANOVA with a Bonferroni post-hoc test was used to assess the changes over time. For non-normal distribution data, statistical significance was analyzed using the Mann–Whitney test between two groups, and the Kruskal–Wallis test with Dunn’s multiple comparisons test for three or more groups. *P* < 0.05 was defined as statistical significance. And significance in all figures is listed as follows: *p < 0.05, **p < 0.01, ***p < 0.001, and ****p < 0.0001 significant vs. control; ^#^*p* < 0.05, ^##^*p* < 0.01, ^###^*p* < 0.001, and ^####^*p* < 0.0001 significant vs. ADRC; *NS*, no significant difference.

## Results

### ADRCs stimulate angiogenesis and transfer mitochondria to endothelial cells and macrophages in hindlimb ischemia mice

**Fig. 1:**
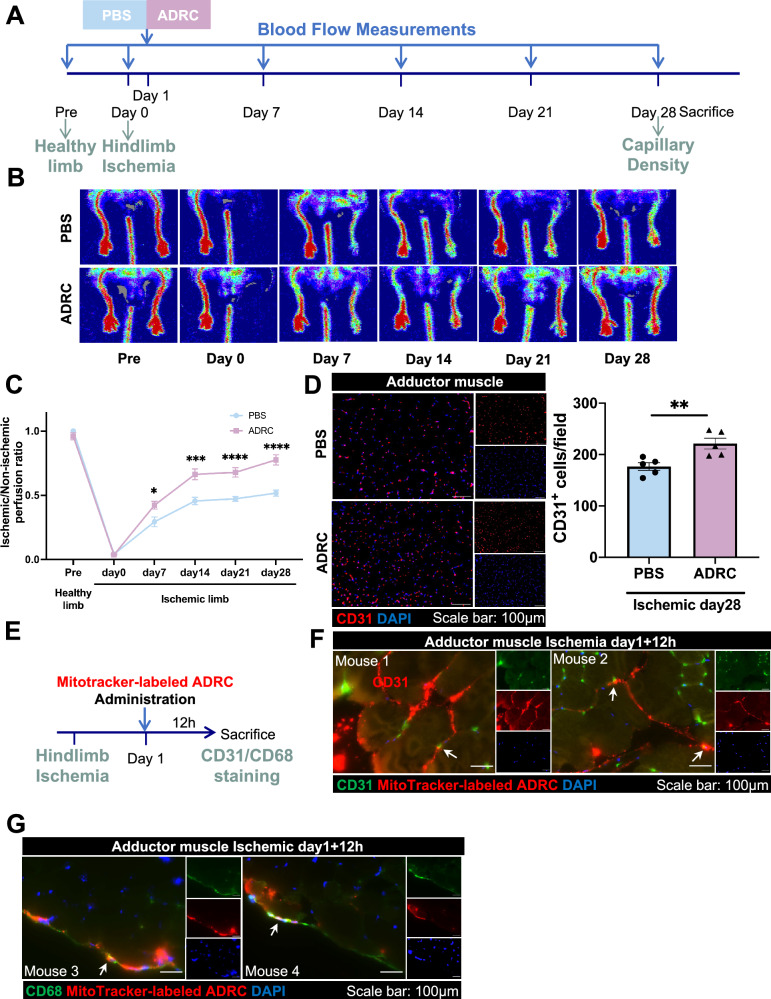
ADRCs transfer mitochondria to both endothelial cells and macrophages, and further promote angiogenesis in the hindlimb ischemia model. **A** Schematic illustration for the administration of PBS and ADRCs, and further therapeutic evaluations in a mouse hindlimb ischemia model. **B** Representative blood perfusion images by Laser Doppler of a hindlimb ischemia model pre-surgery and at ischemic days 0, 7, 14, 21, and 28 in PBS and ADRC group. **C** Quantitate analysis of ischemic/non-ischemic perfusion ratio at different time points (n = 13 per group). **D** Representative adductor muscle images stained by CD31 (red) and DAPI (blue) in the PBS or ADRC group at ischemic day 28. Scale bar represents 100 µm (20 × magnification). Quantification of CD31-positive cells per field at ischemic day 28 under hindlimb ischemia condition (n = 5 per group). **E** Schematic illustration for the administration of Mitotracker red-labeled ADRCs and mitochondrial transfer evaluation in a hindlimb ischemia mouse model. **F** Representative adductor muscle images stained by CD31 (green). The transferred mitochondria (red) originating from ADRCs are labeled with white arrows. Scale bar represents 100 µm (60 × magnification). **G** Representative adductor muscle images stained by CD68 (green). The transferred mitochondria (red) originating from ADRCs are labeled with white arrows. Scale bar represents 100 µm (60 × magnification). Data are shown as mean ± SEM and analyzed by two-way ANOVA with Bonferroni post hoc tests (**C**) or analyzed by unpaired Student t-test (**D**)

First, we investigated the therapeutic angiogenesis with ADRCs using blood perfusion and capillary density in a murine hindlimb ischemia model (Fig. 1A). Blood perfusion measured by Laser Doppler Imaging revealed ADRC implantation promotes angiogenesis significantly at ischemic days 7, 14, 21, and 28 compared with the PBS group (Fig. 1B and C).. Immunostaining using CD31 showed increased capillary density at ischemic day 28 and also demonstrated increased angiogenesis in the ADRC group (Fig. 1D). Following those results, we examined the potential mechanism in ADRC-augmented angiogenesis. Previous studies have shown that mitochondrial transport through intercellular communication could rescue tissue damage and restore physiological function [32–34]. Therefore, to determine whether mitochondria from ADRCs could transfer to other cells in hindlimb ischemia condition, we pre-labeled ADRC with MitoTracker red or Mitochondria-GFP and injected them into the adductor muscle of hindlimb ischemia mice at ischemic day 1 and then further sacrificed after 12 h of injection (Fig. 1E and Figure S1A). Adductor muscle sections showed the mitochondria (red) from MitoTracker red labeled-ADRC or mitochondria (green) from Mitochondria-GFP-labeled ADRC were colocalized with CD31 antibody-labeled endothelial cells (Fig. 1F and Figure S1B) or CD68 antibody-labeled macrophages (Fig. 1G and Figure S1C), indicating the evidence of mitochondrial transfer from ADRCs to endothelial cells and macrophages in hindlimb ischemia mouse model.

### ADRCs could transfer mitochondria to HUVECs at least through Connexin43-based gap junctions and tunneling nanotubes (TNT) in vitro

**Fig. 2:**
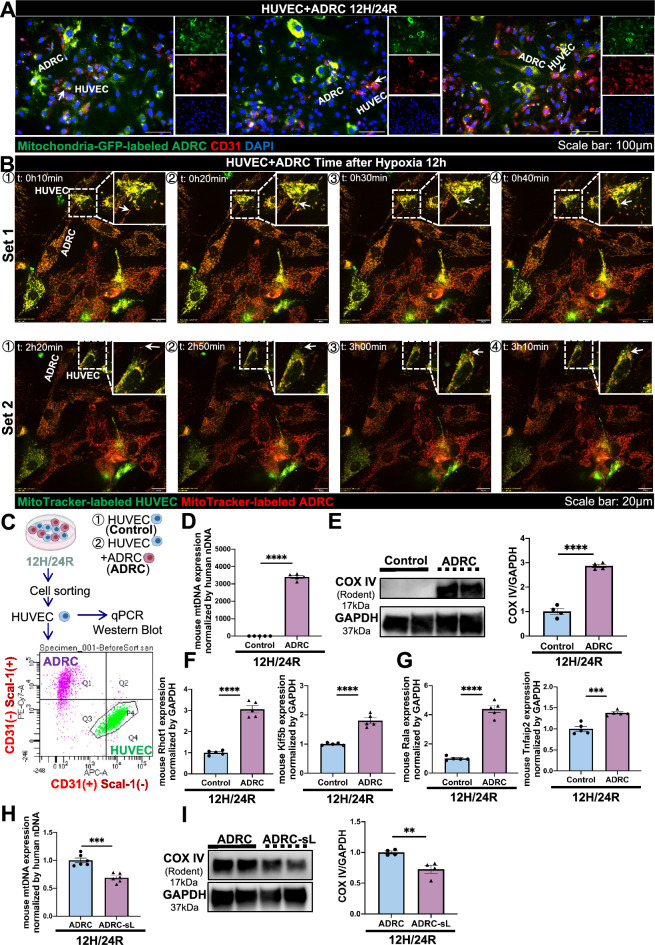
Mitochondrial transfer from ADRCs to HUVECs at least via Connexin43-based gap junctions and tunneling nanotubes (TNT) (nanotubular-like structure). **A** Representative images showing Mitochondria-GFP-labeled ADRC-derived mitochondria (green) within CD31-stained HUVECs after 12 h of hypoxia and 24 h of reoxygenation (12H/24R). White arrows indicate HUVECs with GFP-mitochondria transferred from ADRCs. Scale bar represents 100 µm (20 × magnification). **B** Time-lapse Confocal live-cell images at different time points showing the transfer of ADRC-derived mitochondria (white arrow, red) between MitoTracker Red-labeled ADRCs and MitoTracker Green-labeled HUVECs after 12 h of hypoxia. Scale bar represents 20 µm (60 × magnification). **C** Flow chart of coculture of ADRCs and HUVECs and further evaluation in 12H/24R condition. After 12H/24R, CD31 sorted HUVECs were used for further experiments. **D** Quantification of mouse mtDNA expression in sorted HUVECs. Control indicates HUVEC only group and ADRC indicates sorted HUVECs from the coculture of ADRCs and HUVECs group (n = 5 per group). **E** Representative western blots and quantification of rodent-specific COX IV and GAPDH in sorted HUVECs. Control indicates HUVEC only group and ADRC indicates sorted HUVECs from the coculture of ADRCs and HUVECs group (n = 4 per group). **F** Mouse mRNA expression of microtubule-based markers *Rhot1* and *Kif5b* in control and ADRC group after 12H/24R. Control indicates ADRC only group and ADRC indicates coculture of ADRCs and HUVECs group (n = 5 per group). **G** Mouse mRNA expression of actin-based markers *Rala* and *Tnfaip2* in control and ADRC group after 12H/24R. Control indicates ADRC only group and ADRC indicates the coculture of ADRCs and HUVECs group (n = 5 per group). **H** Quantification of mouse mtDNA expression in sorted HUVECs. ADRC indicates sorted HUVECs from coculture of ADRCs and HUVECs group and ADRC-sL indicates sorted HUVECs from coculture of ADRC-sLs (with siCX43 transfection and Lat A treatment) and HUVECs group (n = 6 per group). **I** Representative western blots and quantification of rodent-specific COX IV and GAPDH in sorted HUVECs. ADRC indicates sorted HUVECs from the coculture of ADRCs and HUVECs group and ADRC-sL indicates sorted HUVECs from the coculture of ADRC-sLs (with siCX43 transfection and Lat A treatment) and HUVECs group (n = 4 per group). Data are shown as mean ± SEM and analyzed by unpaired Student t-test

To comprehensively understand the mitochondrial transfer mechanism of ADRCs, we further examined mitochondrial transfer from live ADRCs to HUVECs in vitro by pre-labeling ADRCs with MitoTracker green and HUVECs with CellTracker orange before coculture under 12H/24R condition. The formation of a nanotubular-like structure (Figure S3A) and connexin43-based gap junction (Figure S2A) were observed. The representative electron microscopy images also showed the TNT structure formed from ADRCs and transferring mitochondria to HUVECs (Figure S2G) after 12H/24R. Real-time live imaging using confocal microscopy also showed the dynamic movement of mitochondria (red) from MitoTracker red-labeled ADRCs to MitoTracker green-labeled HUVECs after 12 h of hypoxia (Fig. 2B and Video S1). In addition, after 12H/24R condition, mitochondria (green) from Mitochondria-GFP-labeled ADRC was also successfully transferred to HUVECs (Fig. 2A). To further confirm mitochondrial transfer from ADRCs to HUVECs, we use FACS-sorted HUVECs after cocultured with ADRCs for further analysis (Fig. 2C). qPCR and western blot analysis revealed mouse mitochondria DNA (mtDNA) and mouse-derived COX IV protein were detected in sorted-HUVECs, also indicating successful transfer of mitochondria from ADRCs to HUVECs (Fig. 2D and E). To comprehensively understand the structure modes in the mitochondrial transfer of ADRCS, we planned to inhibit mitochondrial transfer from ADRCs. Connexin43 plays a key role in regulating mitochondrial transfer [35]. Therefore, we first knocked down Connexin43 in ADRCs to confirm the gap junction structure, and the western blot result showed that we successfully knocked down Connexin43 in ADRCs (Figure S2B). But qPCR and western blot results showed that both mouse mtDNA and mouse-derived COX IV protein are upregulated in the ADRC-siCX43 group (Figure S2C and S2D), indicating there are other structures in the mitochondrial transfer mechanism of ADRCs. Since qPCR results of microtubule-based and actin-based TNT markers [36] also confirmed TNT structure in the mitochondrial transfer mechanism of ADRCs (Fig. 2F and G). Therefore, we next inhibited TNT in ADRCs by tunneling nanotubes inhibitor Latrunculin A (Lat A). Even though mtDNA is decreased (Figure S2E), mouse-derived COX IV protein still increased in the ADRC-Lat A group (Figure S2F). Therefore, we used both inhibitions, and mouse mtDNA and mouse-derived COX IV protein were decreased in the ADRC-sL group, confirming that the Connexin43-based gap junctions and TNT structure are both in Mitochondrial transfer modes of ADRCs to HUVECs in vitro (Fig. 2H and I). Our qPCR results also exhibited that mouse mtDNA was detected in HUVECs from a noncontact coculture system, and the mtDNA from ADRCs in HUVECs decreased with the exosome inhibitor GW4869 pretreatment of ADRC, indicating transfer of mitochondria from ADRCs to HUVECs could happen via extracellular vesicles (Figure S7A).

### ADRCs could deliver mitochondria to macrophages in vitro.

**Fig. 3:**
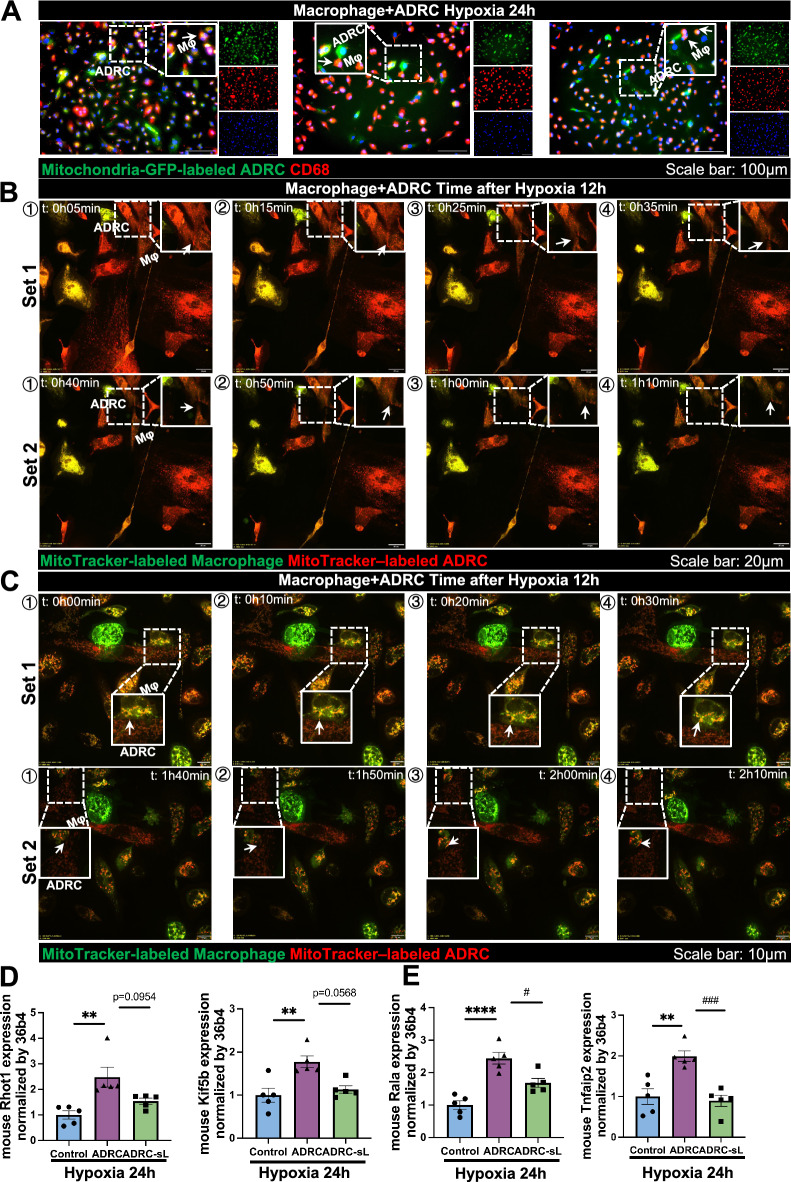
Mitochondrial transfer from ADRC to macrophages. **A** Representative images showing Mitochondria-GFP-labeled ADRC-derived mitochondria (green) within CD68-stained macrophages after 24 h of hypoxia. White arrows indicate macrophages with GFP-mitochondria transferred from ADRCs. Scale bar represents 100 µm (20 × magnification). **B** Time-lapse confocal live-cell images at different time points showing the transfer of ADRC-derived mitochondria (white arrow, red) between MitoTracker Red-labeled ADRCs and MitoTracker Green-labeled macrophages after 12 h of hypoxia. Scale bar represents 20 µm (60 × magnification). **C** Time-lapse confocal live-cell images at different time points showing the transfer of ADRC-derived mitochondria (white arrow, red) between MitoTracker Red-labeled ADRCs and MitoTracker Green-labeled macrophages after 12 h of hypoxia. Scale bar represents 10 µm (100 × magnification). **D** Mouse mRNA expression of microtubule-based markers *Rhot1* and *Kif5b* in control, ADRC, and ADRC-sL group after 24 h of hypoxia. Control indicates macrophage only group, ADRC indicates the coculture of ADRCs and macrophages group, and ADRC-sL indicates the coculture of ADRC-sLs and macrophages group (n = 5 per group). **E** Mouse mRNA expression of actin-based markers *Rala* and *Tnfaip2* in control and ADRC group after 24 h of hypoxia. Control indicates macrophage only group, ADRC indicates the coculture of ADRC and macrophage group, and ADRC-sL indicates the coculture of ADRC-sL and macrophage group (n = 5 per group). Data are shown as mean ± SEM and analyzed by one-way analysis of variance (ANOVA) with Tukey’s multiple comparisons test (**E**) or analyzed by Kruskall-Wallis test with Dunn multiple comparisons test (**D**)

Since local macrophages also contribute to reparative angiogenesis in ischemic tissue [17], we also evaluated mitochondrial transfer from ADRCs to macrophages in vitro by pre-labeling ADRCs with MitoTracker green and macrophages with CellTracker orange before coculture under 24 h of hypoxia condition. The results showed TNT-like structures between ADRCs and macrophages (Figure S3B), and connexin43-based gap junctions were also observed (Figure S2H). Time-lapse live imaging using confocal microscopy also showed dynamic mitochondrial (red) transfer from MitoTracker red-labeled ADRCs to MitoTracker green-labeled macrophages after 12 h of hypoxia (Fig. 3B, C, Video S2, and Video S3). Furthermore, after 24 h of hypoxia, mitochondria (green) from Mitochondria-GFP-labeled ADRC was also successfully transferred to macrophages (Fig. 3A). qPCR result of microtubule-based and actin-based TNT markers further confirmed TNT structure in the mitochondrial transfer mechanism between ADRCs and macrophages (Fig. 3D and E), indicating that mitochondria could transfer from ADRCs to macrophages in vitro at least via the Connexin43-based gap junctions and TNT structure.

### ADRC-transferred mitochondria activate mitochondrial biogenesis and stimulate angiogenic reactions in recipient HUVECs

**Fig. 4:**
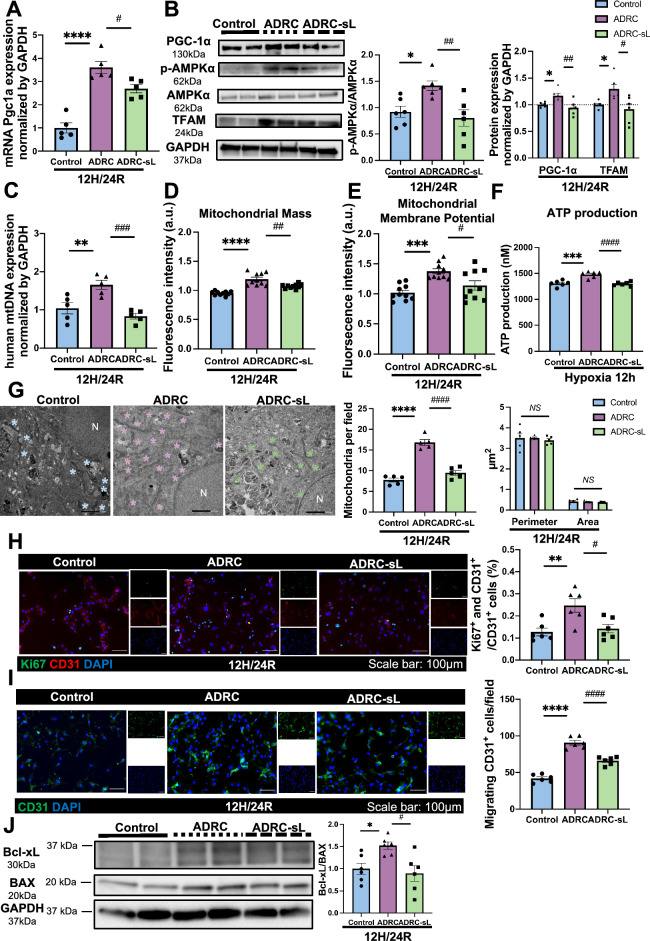
Mitochondrial transfer from ADRC increases mitochondrial biogenesis and angiogenetic ability in HUVEC. **A** Human *Pgc1a* mRNA expression in control, ADRC, and ADRC-sL group after 12H/24R. Control indicates HUVECs only group, ADRC indicates coculture of ADRCs and HUVECs group and ADRC-sL indicates coculture of ADRC-sLs and HUVECs group (n = 5 per group). **B** Representative western blots and quantification of PGC-1α, p-AMPKα, AMPKα, TFAM, and GAPDH in sorted HUVECs after 12H/24R. Control indicates HUVEC only group, ADRC indicates sorted HUVECs from the coculture of ADRCs and HUVECs group and ADRC-sL indicates sorted HUVECs from the coculture of ADRC-sLs and HUVECs group (n = 6 per group). **C** Quantification of human mtDNA expression in sorted HUVECs after 12H/24R. Control indicates HUVEC only group, ADRC indicates sorted HUVECs from coculture of ADRCs and HUVECs group and ADRC-sL indicates sorted HUVECs from coculture of ADRC-sLs and HUVECs group (n = 5 per group). **D** Fluorescence intensity in mitochondrial mass determined by MitoTracker Green in HUVECs after 12H/24R. Control indicates MitoTracker Green-labeled HUVEC only group, ADRC indicates the coculture of ADRCs and MitoTracker Green-labeled HUVECs group and ADRC-sL indicates the coculture of ADRC-sLs and MitoTracker Green-labeled HUVECs group (n = 10 per group). **E** Fluorescence intensity in mitochondrial membrane potential determined by MitoTracker Red in HUVECs after 12H/24R. Control indicates MitoTracker Red-labeled HUVEC only group, ADRC indicates coculture of ADRCs and MitoTracker Red-labeled HUVECs group and ADRC-sL indicates coculture of ADRC-sLs and MitoTracker Red-labeled HUVECs group (n = 10 per group). **F** Changes of intracellular ATP concentration in control, ADRC, and ADRC-sL group after 24 h of hypoxia. Control indicates HUVECs only group, ADRC indicates coculture of ADRCs and HUVECs group and ADRC-sL indicates coculture of ADRC-sLs and HUVECs group (n = 6 per group). **G** Representative electron microscopy images of mitochondria in HUVECs after 12H/24R. Mitochondria were labeled with asterisk, and nuclei were labeled with N. Quantitative analysis of mitochondria number per field of view and measurements of mitochondria perimeter (µm) and area (µm^2^). Control indicates HUVECs only group, ADRC indicates coculture of ADRCs and HUVECs group and ADRC-sL indicates coculture of ADRC-sLs and HUVECs group. Scale bar represents 2 µm (3000 × magnification) (n = 5 per group). **H** Representative images and quantitative analysis of HUVECs proliferation stained by Ki67 (green) and CD31 (red). Scale bar represents 100 µm (20 × magnification) (n = 6 per group). **I** Representative images and quantitative analysis of migrating cells stained by CD31 (green). Images suggest that not only more HUVECs (DAPI co-stained with CD31) but also more ADRCs (DAPI without CD31 staining) migrated to the lower chamber. Scale bar represents 100 µm (20 × magnification) (n = 6 per group). **J** Representative western blots and quantification of Bcl-xL, BAX, and GAPDH in sorted HUVECs after 12H/24R. Control indicates HUVEC only group, ADRC indicates sorted HUVECs from the coculture of ADRCs and HUVECs group and ADRC-sL indicates sorted HUVECs from the coculture of ADRC-sLs and HUVECs group (n = 6 per group). Data are shown as mean ± SEM and analyzed by one-way analysis of variance (ANOVA) with Tukey’s multiple comparisons test (**A**, **B** [protein p-AMPKα/AMPKα, and PGC-1α], **C**–**J**) or analyzed by Kruskall-Wallis test with Dunn multiple comparisons test (**B** [protein TFAM])

Next, we try to understand the effect of ADRC-derived mitochondria on recipient cells. Fluorescence intensity analysis of confocal microscopy time-lapsed images on HUVECs cocultured with ADRCs showed the colocalization between ADRC-derived mitochondria (red) and HUVECs mitochondria (green) (Figure S3C and Video S4), indicating ADRC-derived mitochondria were dynamically colocalized with the mitochondria in HUVECs. Since mitochondrial respiration and glycolysis are essential for endothelial proliferation and angiogenesis [37, 38], we performed the Mito stress test and glycolysis stress test using the Seahorse flux analyzer and found that mitochondrial respiration and glycolysis rates are significantly increased in HUVECs cocultured with ADRCs compared to control HUVECs, and glycolysis capacity is significantly down-regulated in HUVECs cocultured with ADRC-sLs compared to HUVECs cocultured with ADRCs (Figure S8A and B). Those results indicated the positive role of ADRCs in mitochondrial respiration and glycolysis of HUVECs. We then try to determine the following protective mechanism induced by ADRC-derived mitochondria in HUVECs. As many important biological processes in endothelial cells, including energy production, angiogenesis, apoptosis, and the inflammatory response, could be regulated by maintaining mitochondrial homeostasis via the coordination of mitochondrial biogenesis and dynamics [39]. Therefore, we next investigated whether ADRC-transferred mitochondria could rescue hypoxia/reoxygenation-induced damage of HUVEC by mitochondria dynamics and biogenesis mechanism. Since PGC-1α is a key regulator of mitochondrial biogenesis [40], the expression of PGC-1α and its upstream signaling AMPKα together with its downstream factors mitochondrial transcription factor A (TFAM) in sorted HUVEC from cocultures were studied next. Results showed upregulation of phospho-AMPKα, PGC-1α, and TFAM in HUVEC coculture with ADRC, and this effect was partially canceled using inhibition of both Connexin43-based gap junction and TNT of ADRCs (Fig. 4A and B). qPCR result of increased human mtDNA in sorted HUVECs from cocultures (Fig. 4C) and electron microscopy analysis of mitochondria number, perimeter, and area in HUVECs (Fig. 4G) further confirm ADRC-derived mitochondria-induced mitochondrial biogenesis mechanism. The analysis of mitochondrial dynamics showed coculturing with ADRCs didn’t change mitochondria fusion-related markers such as OPA1, MFN1, and MFN2 expression levels in HUVECs (Figure S4A, S4B, S4C, and S4D). Furthermore, quantification of mitochondria area percentage in different categories using an electron microscope showed no differences among the three groups (Figure S4E). Those results suggest inhibition of mitochondria transfer from ADRC to HUVEC did not change the mitochondrial dynamics mechanism in HUVEC. ADRC-transferred mitochondria also induced an increase in mitochondrial mass (Fig. 4D), mitochondrial membrane potential (Fig. 4E), and ATP production (Fig. 4F) of HUVECs. Additionally, in vitro function assay of proliferation and migration ability showed mitochondria transfer from ADRCs to HUVECs could promote angiogenic ability of HUVECs (Fig. 4H and I). Also, the ratio of Bcl-xL/Bax protein expression was upregulated in HUVECs, which indicated that ADRC-derived mitochondria could inhibit apoptosis (Fig. 4J). Collecting together, the results revealed that mitochondria transferred from ADRC could activate AMPKα/PGC-1α/TFAM signaling and induce mitochondrial biogenesis in HUVECs, which further promoted angiogenesis in HUVECs.

### ADRC-transferred mitochondria inhibit inflammatory response in macrophages

**Fig. 5:**
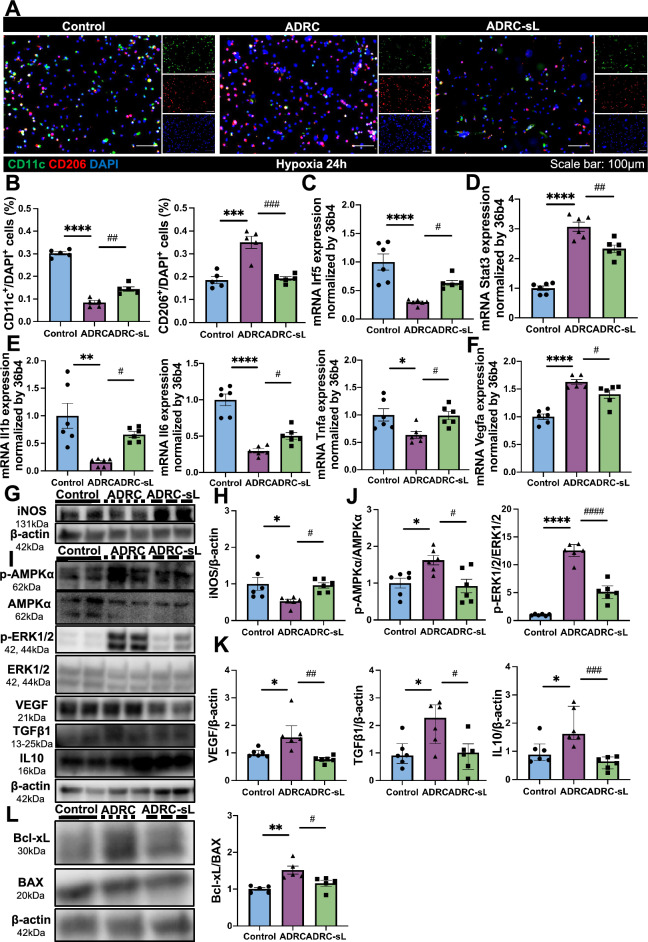
Mitochondrial transfer from ADRC changes macrophages toward an anti-inflammatory phenotype. Control indicates macrophages only group, ADRC indicates coculture of ADRCs and macrophages group and ADRC-sL indicates coculture of ADRC-sLs and macrophages group after 24 h of hypoxia. **A** Representative images and **B** quantitative analysis stained by CD11c (green) and CD206 (red) in the control, ADRC and ADRC-sL group. Scale bar represents 100 µm (20 × magnification) (n = 5 per group). **C** Mouse *Irf5* mRNA expression in control, ADRC and ADRC-sL group (n = 6 per group). **D** Mouse *Stat3* mRNA expression in control, ADRC and ADRC-sL group (n = 6 per group). (E) *Il1b, Il6, and Tnfα* mRNA expression in control, ADRC and ADRC-sL group (n = 6 per group). **F** *Vegfa* mRNA expression in control, ADRC and ADRC-sL group (n = 6 per group). **G** Representative western blots and **H** quantification of iNOS and β-actin in control, ADRC and ADRC-sL group (n = 6 per group). **I** Representative western blots and quantification of **J** p-AMPKα/AMPKα, p-ERK1/2/ ERK1/2, **K** VEGF, TGFβ1, IL10 and β-actin in control, ADRC and ADRC-sL group (n = 6 per group). **L** Representative western blots and quantification of Bcl-xL, BAX and β-actin in control, ADRC and ADRC-sL group (n = 5 per group). Data are shown as mean ± SEM and analyzed by one-way analysis of variance (ANOVA) with Tukey’s multiple comparisons test (**B**–**E** [mRNA *Il6*, and *Tnfα*], **F**–**L**) or analyzed by Kruskall-Wallis test with Dunn multiple comparisons test (**E** [mRNA *Il1b*])

Next, we investigated the effect of mitochondrial transfer from ADRCs on macrophages under hypoxia conditions. Fluorescence intensity analysis of live time confocal microscopy images on macrophages cocultured with ADRCs showed the colocalization between ADRC-derived mitochondria (red) and macrophages mitochondria (green) in macrophages (Figure S3D and Video S5), showing ADRC-derived mitochondria were highly colocalized with the mitochondria in macrophages. We then studied the underlying mechanism induced by transferred mitochondria from ADRCs in macrophages. In vitro coculture study by immunofluorescence showed that mitochondria transferred from ADRCs suppressed CD11c^+^ M1 pro-inflammatory macrophages and enhanced CD206^+^ anti-inflammatory M2 macrophages (Fig. 5A and B). Furthermore, we tested signaling molecules involved in macrophage M1/M2 polarization using qPCR and western blot [41]. qPCR result showed *Irf5* mRNA which driven M1 polarization was downregulated and *Stat3* mRNA which driven M2 polarization was upregulated in the ADRC group (Fig. 5C and D). qPCR result of pro-inflammatory cytokines, such as *Il1b*, *Il6*, and *Tnfα* mRNA expression was also decreased, and M2-associated markers *Vegfa* mRNA expression was increased in the ADRC group (Fig. 5E and F). Also, M1 markers iNos protein expression was inhibited, and M2-related signaling and markers p-AMPKα, p-ERK1/2, VEGF, TGFβ1, and IL10 protein expression was promoted in the ADRC group (Fig. 5G–K), which further confirmed ADRC-transferred mitochondria induce macrophages toward anti-inflammatory phenotype. The effect of mitochondrial transfer from ADRC on macrophage apoptosis was detected by Bcl-xL/Bax protein expression ratio, and the down-regulation ratio in the ADRC group indicated that ADRC-derived mitochondria could inhibit apoptosis (Fig. 5L). Together, the above data suggested mitochondrial transfer from ADRCs acts on macrophage polarization to prevent M1 pro-inflammatory markers and augment M2 anti-inflammatory markers.

### Partial inhibition of mitochondrial transfer from ADRCs abrogates angiogenesis in hindlimb ischemia

**Fig. 6:**
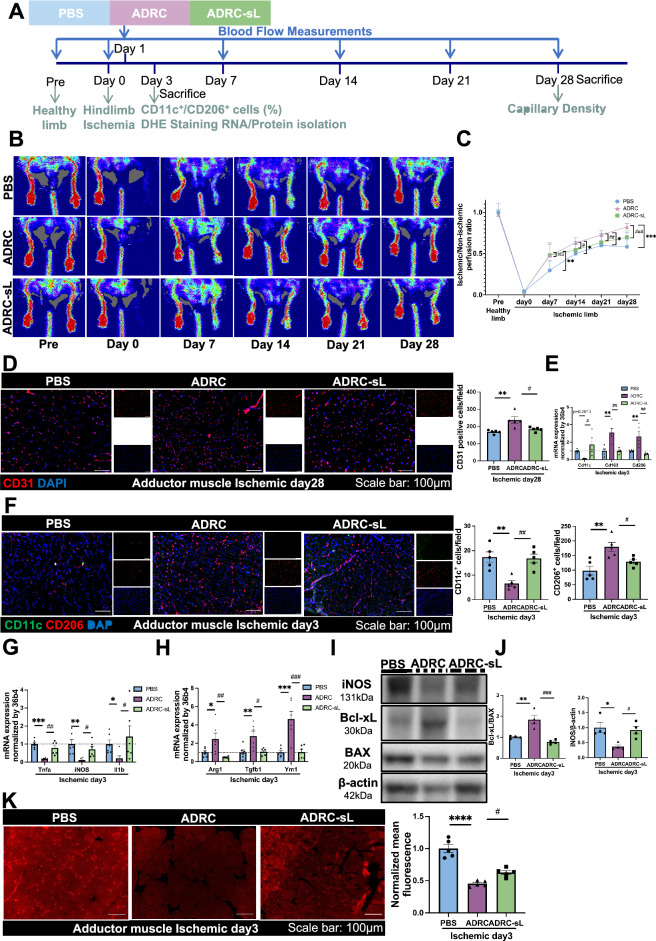
Blockade of mitochondrial transfer from ADRC impedes angiogenesis in hindlimb ischemia model. **A** Schematic illustration for the administration of ADRCs and ADRC-sLs and further therapeutic evaluations in mouse hindlimb ischemia model. **B** Representative blood perfusion images by Laser Doppler of hindlimb ischemia model pre-surgery and at ischemic day 0, 7, 14, 21, 28 in PBS, ADRC and ADRC-sL group. **C** Quantitate analysis of ischemic/non-ischemic perfusion ratio at different time point (n = 14 per group). **D** Representative adductor muscle images stained by CD31 (red) and DAPI (blue) in PBS, ADRC and ADRC-sL group at ischemic day 28. Scale bar represents 100 µm (20 × magnification). And quantification of CD31 positive cells per field at ischemic day 28 under hindlimb ischemia condition (n = 5 per group). **E** *Cd11c, Cd163, and Cd206* mRNA expression in PBS, ADRC and ADRC-sL group (n = 5 per group). **F** Representative images and quantitative analysis stained by CD11c (green), CD206 (red) and DAPI (blue) in PBS, ADRC and ADRC-sL group. Scale bar represents 100 µm (20 × magnification) (n = 5 per group). **G** *Tnfα, iNos, and Il1b* mRNA expression in PBS, ADRC and ADRC-sL group (n = 6 per group). **H** *Arg1, Tgfb1, and Ym1* mRNA expression in PBS, ADRC and ADRC-sL group (n = 6 per group). **I** Representative western blots and **J** quantification of iNOS, Bcl-xL, BAX and β-actin in PBS, ADRC and ADRC-sL group (n = 4 per group). **K** Representative images and quantification of dihydroethidium (DHE) staining of adductor muscles in PBS, ADRC and ADRC-sL group. Data are shown as mean ± SEM and analyzed by two-way ANOVA with Bonferroni post hoc tests (**C**), or one-way analysis of variance (ANOVA) with Tukey’s multiple comparisons test (**D**–**G** [mRNA *Tnfα*], **H**, **J** [protein Bcl-xL/BAX] and K) or analyzed by Kruskall-Wallis test with Dunn multiple comparisons test (**G** [mRNA *iNos*, and *Il1b*] and J [protein iNOS])

To further verify the mitochondrial transfer mechanism of ADRCs on therapeutic angiogenesis, we finally treated hindlimb ischemia mice with ADRCs or both Connexin43-knocked down and Lat A treatment of ADRCs at ischemic day 1 (Fig. 6A). H&E results revealed that the muscle fiber size in the ADRC-sL group was significantly lower, and interstitial space was significantly bigger in the adductor muscle compared to the ADRC group (Figure S5B). These changes suggested that pro-angiogenic effect via mitochondrial transfer from ADRCs could ameliorate fiber atrophy of the ischemic hindlimb [42, 43]. The Laser Doppler perfusion Images and CD31 staining at ischemic day 28 showed that Inhibition of mitochondrial transfer from ADRC could attenuate blood perfusion at ischemic days 14, 21, 28 and capillary density (Fig. 6B–D). Also, both qPCR results of macrophage surface markers and immunofluorescence staining with CD11c^+^ (green) and CD206^+^ (red) showed that mitochondria transferred from ADRCs promotes macrophage M2 polarization in a mouse HLI model (Fig. 6E and F). Furthermore, the pro-inflammatory cytokines *Tnfα*, *iNos*, and *Il1b* mRNA expression were suppressed and M2 macrophage-associated markers *Arg1*, *Tgfb1*, and *Ym1* mRNA expression were increased in the ADRC group compared with the PBS group (Fig. 6G and 6H). In addition, the iNos protein expression was also decreased and Bcl-xL/Bax protein expression was increased in the ADRC group compared with the PBS group, also indicating the anti-inflammatory and anti-apoptosis effect in the mitochondrial transfer mechanism of ADRCs (Fig. 6I and J). Furthermore, TUNEL staining also showed ADRC could exhibit anti-apoptosis impact on endothelial cells and macrophages through the mitochondrial transfer mechanism (Figure S9A and S9B). Finally, oxidative fluorescence images using DHE staining showed that mitochondria transfer from ADRCs could reduce superoxide production after tissue ischemia at ischemic day 3 (Fig. 6K).

## Discussion

The major findings of the present study are as follows. (1) Implanted ADRCs can transfer mitochondria to endothelial cells and macrophages and increase angiogenesis in a mouse hindlimb ischemia model. (2) ADRCs can transfer mitochondria to HUVECs and macrophages in vitro through the Connexin43-based gap junctions and tunneling nanotube pathway. (3) Mitochondrial transfer from ADRCs enhances mitochondrial biogenesis and angiogenesis ability of endothelial cells in vitro. (4) Mitochondrial transfer from ADRCs attenuates inflammatory reactions in macrophages in vitro. (5) Inhibition of mitochondrial transfer from ADRCs partially canceled ADRC-induced reparative angiogenesis in a mouse hind limb ischemia model.

MSCs are a promising cell source for damaged tissue regeneration. They exist in bone marrow and adipose tissue. Although previous studies have shown the angiogenesis ability of BM-MSCs [44], ADRCs considered to be less invasive and easier to collect in large quantities than bone marrow-derived MSCs with almost the same potential. In addition, our findings demonstrated that both ADRCs and BM-MSCs significantly enhance the proliferation of HUVECs (Figure S6A). Notably, ADRCs exhibited a superior capacity to promote the migratory potential of HUVECs compared to BM-MSCs (Figure S6B). Furthermore, our group has already clinically introduced therapeutic angiogenesis using ADRCs, which aims to promote angiogenesis for critical limb ischemia and the recovery of blood perfusion [45–47]. The results of the multicenter study (UMIN000010143, jRCTb040190118) have demonstrated therapeutic safety (no adverse events during the perioperative and 6-month observation period) and feasibility [47]. In those cases, we sometimes observed remarkable improvements that exceeded our expectations. Therefore, we speculate from clinical experience that there are other therapeutic mechanisms underlying this therapy. The paracrine effects of ADRCs (secretion of angiogenesis-promoting factors such as SDF1 [11], VEGF-C [5], and PGE2 [17], and exosome-mediated mechanisms, such as microRNA [20, 48]) have been previously investigated in terms of the mechanisms underlying the protective effects of ADRC-based therapy. Recently, mesenchymal stem cells (MSCs) have been reported to exert their functions through intercellular signal communication by providing mitochondria to target cells and transferring them between cells (aka. mitochondrial transfer). The current study provides a perspective for understanding the mitochondrial transfer mechanism of ADRCs that protects against hind limb ischemia in a mouse model.

Intercellular mitochondrial transfer can occur via a variety of modes, such as extracellular vesicles, cell fusion, gap junctions, and TNTs [49, 50]. Depending on the cell type, TNT contain cytoskeletal components, such as actin and microtubules [51]. Furthermore, Connexin 43 is essential for gap junction channel development that controls mitochondrial transfer [52]. Moreover, recipient cells can absorb extracellular vesicles containing mitochondria that have been released from donor cells. The mitochondria can also be exchanged through the fusion of two cells. Transfer of mitochondria from donor stem cells could be triggered by cues, including mitochondrial DNA (mtDNA), ROS, NADPH oxidase-2 (NOX2), Ca2 + , and CD38, from the local microenvironment of an injured recipient cell [53]. Our study showed both in vitro and in a hindlimb ischemia model in vivo, in which mitochondria could be transferred from ADRC to endothelial cells and macrophages. In addition, we demonstrated that the mitochondrial transfer modes of ADRC to HUVEC in vitro occurred at least through the TNT, Connexin43-based gap junctions, and extracellular vesicles, and ADRCs could transfer mitochondria to macrophages at least through the TNT and Connexin43-based gap junctions in vitro.

In the context of transferred mitochondrial kinetics in recipient cells, our results showed that ADRC-derived exogenous mitochondria dynamically co-localized with endogenous mitochondria in both HUVECs and macrophages (Video S4 and S5). Based on previous studies, one of the potential processes could be that the donor mitochondria fuse with the recipient cell mitochondria, thereby rescuing the recipient cell. In addition, the recipient cell could also discard the damaged mitochondria and instead use the given mitochondria for energy generation [54]. Further grasp of these limitations could help facilitate MSC mitochondrial transfer treatment in clinical practice.

Furthermore, we studied the protective mechanism of mitochondrial transfer from ADRCs to recipient cells (both endothelial cells and macrophages). It has been demonstrated that in endothelial cells, transferred mitochondria could lead to improvements in apoptosis, cellular metabolism, ATP level, cell proliferation, mitochondrial respiration, and homeostasis [9, 55, 56]. Additionally, mitochondrial homeostasis is maintained through the coordination of mitochondrial dynamics and mitochondrial biogenesis [57, 58]. Many crucial biological activities, including energy production, angiogenesis, apoptosis, and inflammatory response, could be regulated through retaining mitochondrial homeostasis [59, 60]. Changes in mitochondrial dynamics are under the control of fission (DRP1 and Fission1) and fusion (Mitofusion1, Mitofusion2, and OPA1) proteins, and mitochondrial biogenesis is regulated through PGC-1α via phosphorylation of AMPKα and TFAM proteins [61, 62]. Therefore, we investigated whether ADRC-transferred mitochondria could rescue hypoxia/reoxygenation-induced damage of HUVEC by mitochondria dynamics and biogenesis mechanism and following ATP production, apoptosis, and angiogenesis. The results showed that the mitochondrial transfer mechanism of ADRCs could induce mitochondrial biogenesis in endothelial cells, upregulate ATP production, reduce apoptosis, and promote the angiogenic ability of endothelial cells.

Macrophages are phagocytic cells of the innate immune system that perform various functions in immunological responses and homeostasis. Previous research has demonstrated that macrophages can switch between M1 and M2 polarization and thus exhibit pro- or anti-inflammatory effects [63]. Additionally, research has demonstrated that MSCs can promote tissue repair and reduce inflammation by altering the polarization of macrophages, and studies have proved that MSCs could transfer mitochondria to macrophages therefore promoting their phagocytosis [10]. In the concept of tissue homeostasis and damage repair in MSCs-macrophage crosstalk, we further studied the effect of the mitochondrial transfer mechanism of ADRCs on macrophages in vitro. Our results showed that mitochondrial transfer from ADRCs could directly shift macrophages from the M1- to the M2-phenotype. Next, we investigated the signaling pathways involved in this phenomenon. Previous studies have shown that M1 macrophage polarization could be activated by the IRF5 and promote the expression of pro-inflammatory cytokines, such as IL-1β, IL-6, and TNF-α [64, 65] and indirectly regulate iNos production [66]. In this study, we demonstrated that partially canceling mitochondrial transfer could up-regulate IRF5 signaling and inflammatory cytokines. In M2 polarization, AMPK is a direct upstream signaling protein that initiates anti-inflammatory signaling pathways in macrophages [67, 68]. It also plays an important role in activating IL-10 during STAT3-mediated macrophage M2 polarization [69]. In addition, the ERK signaling pathway has been extensively explored for its role in stimulating angiogenesis, cell growth, and proliferation via phosphorylation responses [70]. Macrophages have been well studied in angiogenesis, too [71]. Studies have also proven that MSCs could reduce apoptosis and promote macrophage M2 polarization [72–74]. This study further demonstrated the above signaling in the mitochondrial transfer mechanism between ADRCs and macrophages interaction under ischemic conditions mimicking the ischemic limbs. In addition, our study indicates that mitochondrial transfer to macrophages shifts macrophage polarization from the M1- to M2-phenotype, then further decreasing inflammation and apoptosis and contributing to reparative angiogenesis.

There are several limitations in our current study. First, we tested C57BL6J mice that are between 8- to10-weeks-old [11, 29, 75], in accordance with previous reports. However, the major angiogenetic problem often happens in people with aging. In the future, this study should be investigated in aged mice as well. Secondly, it remains uncertain whether the observed alterations in gene and protein expression associated with M1/M2 polarization are specific to macrophages within the coculture system comprising ADRCs and peritoneal macrophages, as depicted in Fig. 5C–L. Although the data presented in Supplemental Fig. 10 suggest that these changes occur independently of the expression profiles in ADRCs, further investigation is warranted. Specifically, future studies should involve the isolation of macrophages post-coculture, potentially through cell sorting techniques, followed by comprehensive gene and protein expression analyses to substantiate these findings.

In conclusion, ADRCs can transfer mitochondria to endothelial cells and macrophages under ischemic conditions, at least through gap junction and tunneling nanotube pathways. Internalization of the transferred mitochondria could improve mitochondrial biogenesis in endothelial cells and induce M2 polarization in macrophages, both of which would contribute to reparative angiogenesis. Our findings revealed that mitochondrial transfer could be a potential mechanism for therapeutic angiogenesis by ADRCs.

## Supplementary Information

Below is the link to the electronic supplementary material.

Supplementary file1 (PDF 736 kb)

**Supplemental Fig. 1** Mitochondrial transfer from Mitochondria-GFP-labeled ADRC in a hindlimb ischemia mouse model. (A) Schematic illustration for the administration of Mitochondria-GFP-labeled ADRCs and mitochondrial transfer evaluation in a hindlimb ischemia mouse model. (B) Representative adductor muscle images stained by CD31 (red). The transferred mitochondria (green) originating from ADRCs are labeled with white arrows. Scale bar represents 100µm (20x magnification). (C) Representative adductor muscle images stained by CD68 (red). The transferred mitochondria (green) originating from ADRCs are labeled with white arrows. Scale bar represents 100µm (20x magnification); **Supplemental Fig. 2** Different modes of mitochondrial transfer from ADRC to HUVEC. (A) Representative images of ADRCs coculturing with HUVECs stained by Connexin43 (green) and WGA (red) after 12H/24R. Scale bar represents 100µm (20x magnification). (B) Representative western blots and quantification of Connexin43 and GAPDH in ADRC-scr and ADRC-siCX43 (n=6 per group). (C) Quantification of mouse mtDNA expression in in PBS, ADRC and ADRC-siCX43 group after 12H/24R. Control indicates HUVEC only group, ADRC indicates sorted HUVECs from coculture of ADRCs and HUVECs group and ADRC-siCX43 indicates sorted HUVECs from coculture of ADRCs (with siCX43 transfection) and HUVECs group (n=5 per group). (D) Representative western blots of rodent-specific COX IV and GAPDH in PBS, ADRC and ADRC-siCX43 group after 12H/24R. (E) Quantification of mouse mtDNA expression in in PBS, ADRC and ADRC-Lat A group after 12H/24R. Control indicates HUVEC only group, ADRC indicates sorted HUVECs from coculture of ADRCs and HUVECs group and ADRC-siCX43 indicates sorted HUVECs from coculture of ADRCs (with Lat-A treatment) and HUVECs group (n=5 per group). (F) Representative western blots of rodent-specific COX IV and GAPDH in PBS, ADRC and ADRC-Lat A group after 12H/24R. (G) Representative electron microscopy images of mitochondria transfer structure after 12H/24R. The tube-like structure from ADRC to HUVEC are labeled with white arrows. (H) Representative images of ADRCs coculturing with macrophages stained by Connexin43 (green) and WGA (red) after 24 hours of hypoxia. Scale bar represents 100µm (20x magnification). Data are shown as mean ± SEM and were analyzed by unpaired Student’s t-test (B and E) or analyzed by Mann–Whitney test (C); **Supplemental Fig. 3** Colocalization of ADRC-derived mitochondria in HUVECs and macrophages. (A) Representative live-cell images showing MitoTracker Green-labeled ADRC-derived mitochondria within CellTracker Orange-labeled HUVECs after 12 hours of hypoxia and 24 hours of reoxygenation (12H/24R). White arrows indicate the connection between ADRCs and HUVECs. Scale bar represents 100µm (20x magnification). (B) Representative live-cell images showing MitoTracker Green-labeled ADRC-derived mitochondria within CellTracker Orange-labeled macrophages after 24 hours of hypoxia. White arrows indicate the connection between ADRCs and macrophages. Scale bar represents 100µm (20x magnification). (C) Confocal live-cell images showing colocalization of MitoTracker Red-labeled ADRC-derived mitochondria (red) and MitoTracker Green-labeled HUVECs mitochondria after 12 hours of hypoxia. And fluorescence intensity analysis showing the colocalization. Scale bar represents 20µm (60x magnification). (D) Confocal live-cell images showing colocalization of MitoTracker Red-labeled ADRC-derived mitochondria (red) and MitoTracker Green-labeled Macrophages mitochondria after 12 hours of hypoxia. And fluorescence intensity analysis showing the colocalization. Scale bar represents 10µm (100x magnification); **Supplemental Fig. 4** Mitochondrial dynamics mechanism in sorted HUVECs cocultured with ADRC. (A) Human *Drp1, Fis1, Opa1, Mfn1 and Mfn2* mRNA expression in Control, ADRC and ADRC-sL group. Control indicates HUVECs only group, ADRC indicates coculture of ADRCs and HUVECs group and ADRC-sL indicates coculture of ADRC-sLs and HUVECs group (n=5 per group). (B) Representative western blots and quantification of (C) p-DRP1, DRP1, Fission1, (D) OPA1, Mitofusion1, Mitofusion2 and GAPDH in PBS, ADRC and ADRC-sL group after 12H/24R (n=6 per group). (E) Percentage of mitochondria area that classified as three sizes: < 0.6 μm^2^, 0.6 μm^2^–1.0 μm^2^, and >1.0 μm^2^. Data are shown as mean ± SEM and analyzed by one-way analysis of variance (ANOVA) with Tukey’s multiple comparisons test (A [mRNA *Drp1, Fis1, Mfn1, *and* Mfn2*], C, D [protein Mitofusion1 and Mitofusion2] and E) or analyzed by Kruskall-Wallis test with Dunn multiple comparisons test (A [mRNA *Opa1*] and D [protein OPA1]); **Supplemental Fig. 5** Histological changes in a mouse hindlimb ischemia model at ischemic day 21 and day 28. (A) Representative hindlimb photographs of sham, PBS, ADRC, and ADRC-sL group at ischemic day 21 and day 28. (B) Representative adductor muscle sections stained with H&E of sham, PBS, ADRC, and ADRC-sL group at ischemic day 21 and day 28. Scale bar represents 100µm (20x magnification). And quantification of muscle fiber size (n=4 per group). Data are shown as mean ± SEM and analyzed by one-way analysis of variance (ANOVA) with Tukey’s multiple comparisons test; **Supplemental Fig. 6** The effect of ADRCs and BMMSCs on the angiogenesis ability of HUVECs under 12H/24R condition. (A) Representative images and quantitative analysis of HUVECs proliferation stained by Ki67 (green) and CD31 (red). Scale bar represents 100µm (20x magnification) (n=4 per group). (B) Representative images and quantitative analysis of migrating cells stained by CD31 (green). Scale bar represents 100µm (20x magnification) (n=4 per group). Data are shown as mean ± SEM and analyzed by one-way analysis of variance (ANOVA) with Tukey’s multiple comparisons test; **Supplemental Fig. 7** Mitochondria could transfer from ADRCs to HUVECs with no direct contact under 12H/24R condition. (A) Non-direct contact system with transwell insert to evaluate mitochondrial transfer from ADRCs to HUVECs. Quantification of mouse mtDNA expression in HUVECs. (n=5 per group). Data are shown as mean ± SEM and were analyzed by one-way analysis of variance (ANOVA) with Tukey’s multiple comparisons test; **Supplemental Fig. 8** Metabolism in HUVECs and HUVECs cocultured with ADRC under 12H/24R condition. (A) Mito stress test results in ADRC, HUVEC, and HUVEC+ADRC (n=6 per group). (B) Glycolysis stress test results in HUVEC, HUVEC+ADRC, and HUVEC+ADRC-sL (n=6 per group). Data are shown as mean ± SEM and analyzed by one-way analysis of variance (ANOVA) with Tukey’s multiple comparisons test (A [Basal Respiration] and B) or analyzed by Kruskall-Wallis test with Dunn multiple comparisons test (A [ATP Production]); **Supplemental Fig. 9** Mitochondrial transfer from ADRC decreases apoptotic cells in a mouse hindlimb ischemia model at ischemic day 3. (A) Representative images and quantitative analysis stained by TUNEL (green), CD31 (red) and DAPI (blue) in PBS, ADRC and ADRC-sL group. Scale bar represents 100µm (20x magnification) (n=5 per group). (B) Representative images and quantitative analysis stained by TUNEL (green), CD68 (red) and DAPI (blue) in PBS, ADRC and ADRC-sL group. Scale bar represents 100µm (20x magnification) (n=5 per group). Data are shown as mean ± SEM and analyzed by one-way analysis of variance (ANOVA) with Tukey’s multiple comparisons test (A and B); **Supplemental Fig. 10** M1 and M2 macrophages-related genes expression in macrophages and ADRCs in 24h of hypoxia condition. (A) Mouse *Irf5* mRNA expression in Macrophage and ADRC group (n=5 per group). (B) Mouse *Stat3* mRNA expression in Macrophage and ADRC group (n=5 per group). (C) *Il1b, Il6, and Tnfα* mRNA expression in Macrophage and ADRC group (n=5 per group). (D) *Vegfa* mRNA expression in Macrophage and ADRC group (n=5 per group). (E) Representative western blot images of iNOS, p-AMPKα, AMPKα, p-ERK1/2, ERK1/2, Bcl-xL, BAX, VEGF, TGFβ1, IL10 and β-actin in Macrophage and ADRC group. (F) Quantification of iNOS and β-actin, (G) p-AMPKα/AMPKα and p-ERK1/2/ ERK1/2, (H) VEGF, TGFβ1, IL10 and β-actin, and (I) Bcl-xL/BAX in Macrophage and ADRC group (n=6 per group). Data are shown as mean ± SEM and were analyzed by unpaired Student’s t-test (A, B, C, D, F, G, H [TGFβ1, IL10] and I) or analyzed by Mann–Whitney test (H [VEGF])

Supplementary Movie S1: Dynamics movements of mitochondrial transfer from MitoTracker red-labeled ADRCs to MitoTracker green-labeled HUVECs after 12h of hypoxia (Every 5 min, 60x magnification).

Supplementary Movie S2: Dynamics movements of mitochondrial transfer from MitoTracker red-labeled ADRCs to MitoTracker green-labeled macrophages after 12h of hypoxia (Every 5 min, 60x magnification).

Supplementary Movie S3: Dynamics movements of mitochondrial transfer from MitoTracker red-labeled ADRCs to MitoTracker green-labeled macrophages after 12h of hypoxia (Every 5 min, 100x magnification).

Supplementary Movie S4: Fluorescence intensity analysis of confocal microscopy time-lapsed images on HUVECs cocultured with ADRCs (Every 20 min).

Supplementary Movie S5: Fluorescence intensity analysis of confocal microscopy time-lapsed images on macrophages cocultured with ADRCs (Every 20 min).

## Data Availability

No datasets were generated or analysed during the current study.

## Abbreviations

ADRCs: Adipose-derived regenerative cells
DAPI: 4′,6-Diamidino-2-phenylindole
HLI: Hindlimb ischemia
HUVEC: Human umbilical vein endothelia cell
LDPI: Laser Doppler perfusion imaging
qPCR: Quantitative polymerase chain reaction
ROS: Reactive oxygen species
