# Supplementary material for: Mitochondrial transfer from adipose-derived regenerative cells contributes therapeutic angiogenesis in a murine hindlimb ischemia model
Source: Angiogenesis. 2025 Sep 10;28(4):49. doi: 10.1007/s10456-025-10001-z (PMC12423147; doi:10.1007/s10456-025-10001-z)

Mitochondrial transfer of adipose-derived regenerative cells contributes therapeutic angiogenesis in a murine hindlimb ischemia model.

Yiyang Che, M.D.[1]; Yuuki Shimizu, M.D., Ph.D.[1*]; Takumi Hayashi, M.D.[1]; Junya Suzuki, M.D., Ph.D.[1]; Zhongyue Pu, M.D., Ph.D.[1]; Kazuhito Tsuzuki, M.D., Ph.D.[1]; Shingo Narita, M.D., Ph.D.[1]; Yoshimitsu Yura, M.D., Ph.D.[1]; Rei Shibata, M.D., Ph.D.[2]; Toyoaki Murohara, M.D., Ph.D.[1]

[1]Department of Cardiology, Nagoya University Graduate School of Medicine, Nagoya 466-8550, Japan
[2]Department of Advanced Cardiovascular Therapeutics, Nagoya University Graduate School of Medicine, Nagoya 466-8550, Japan

Figure 2E

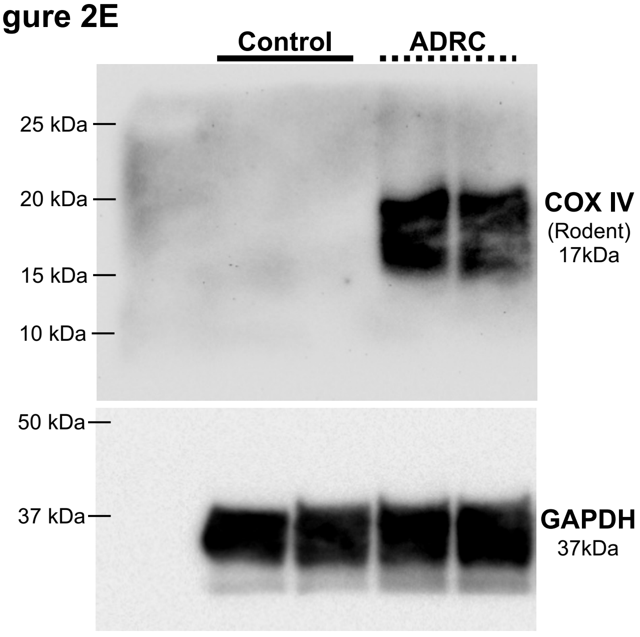

Figure 2I

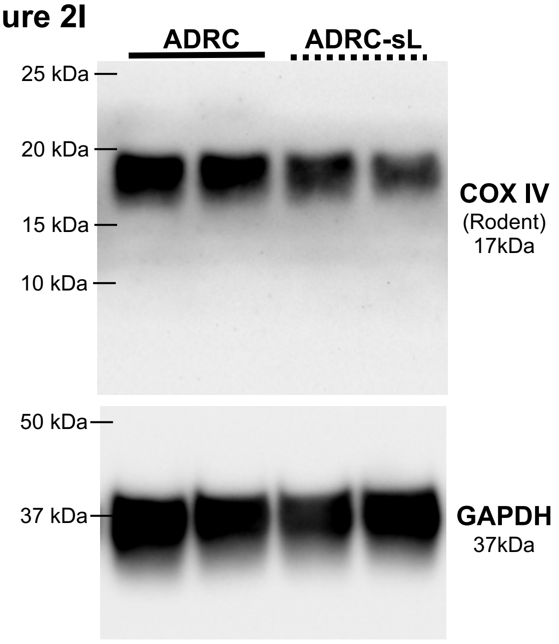

Figure 4B

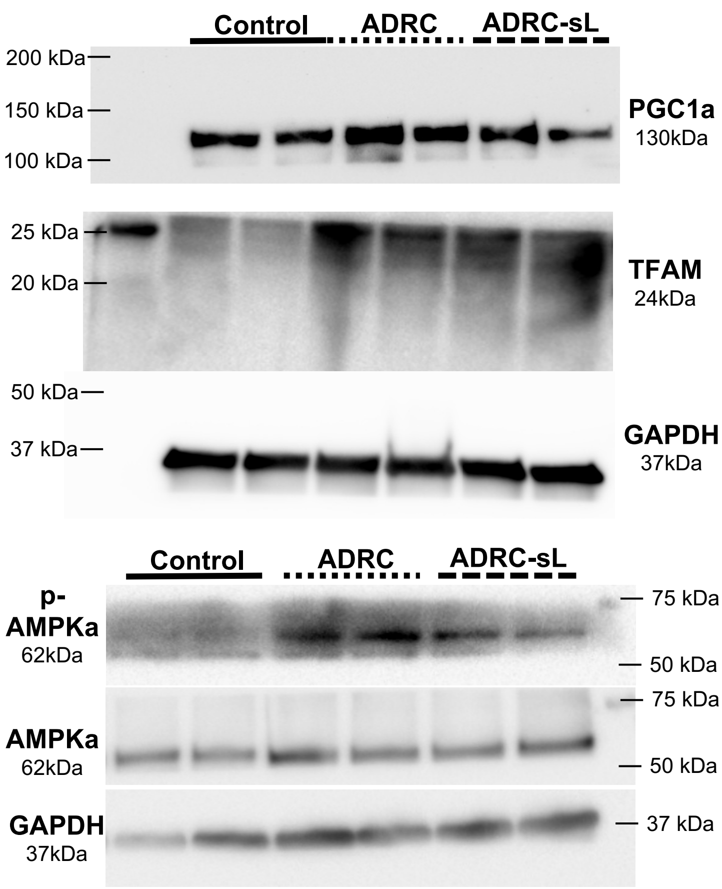

Figure 4J

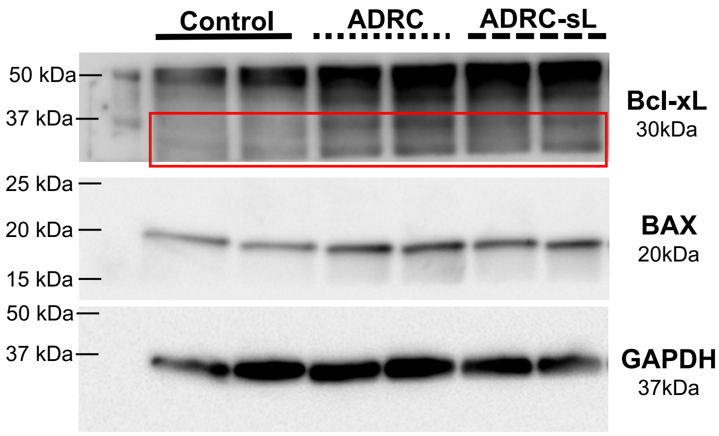

Figure 5G

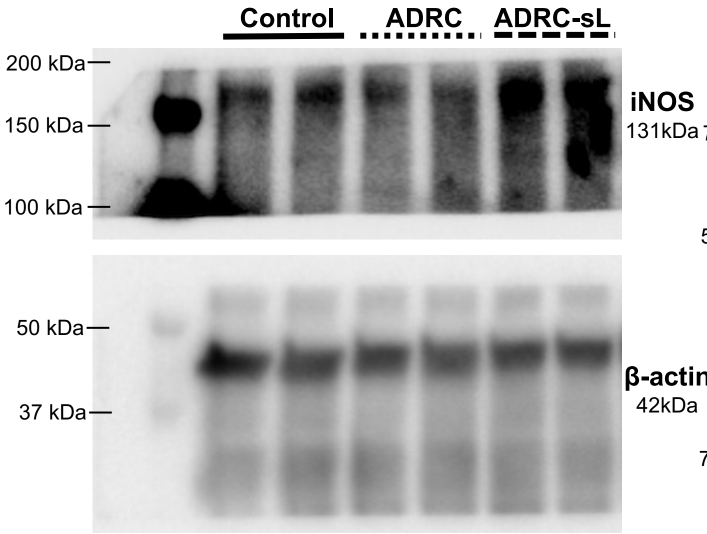

Figure 5I (set 1)

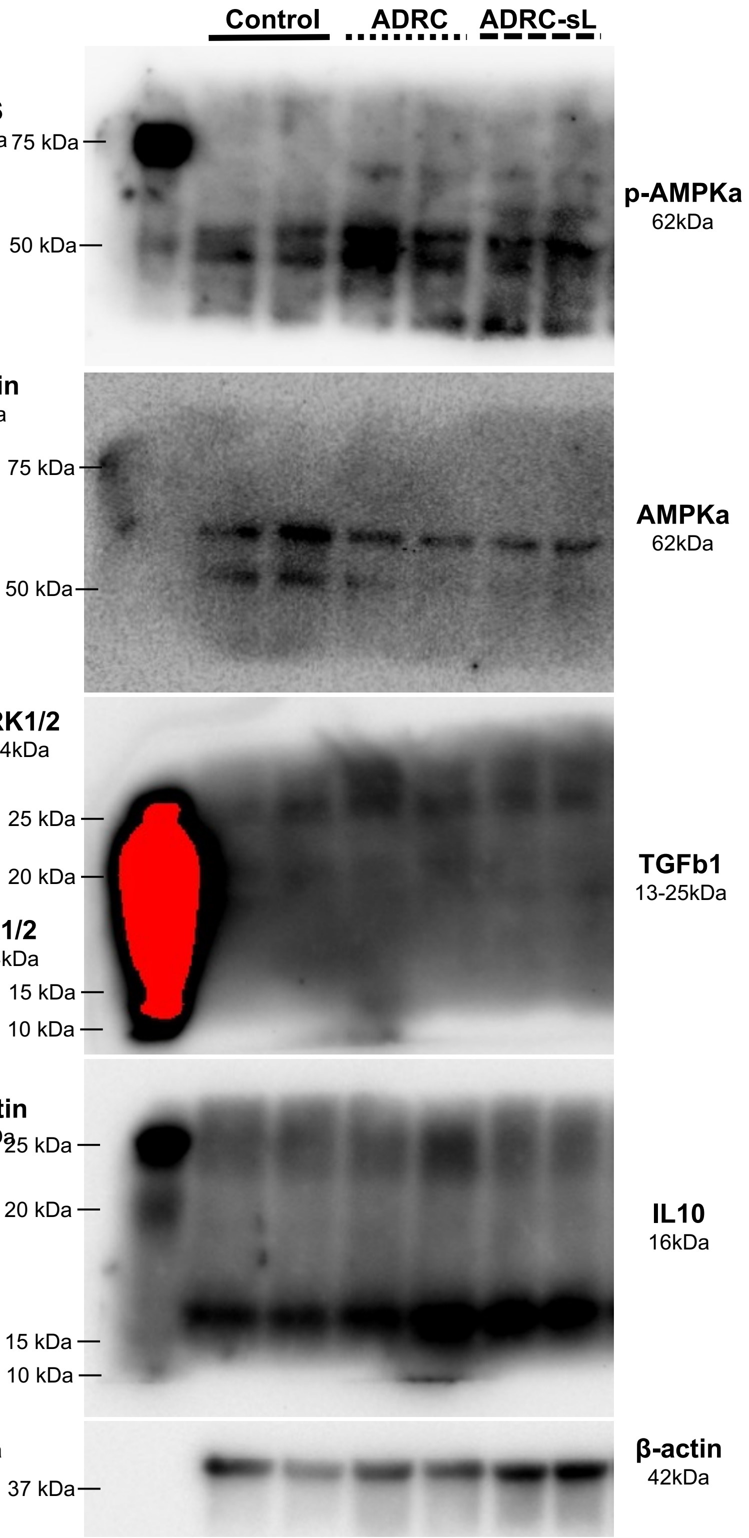

Figure 5I (set 2)

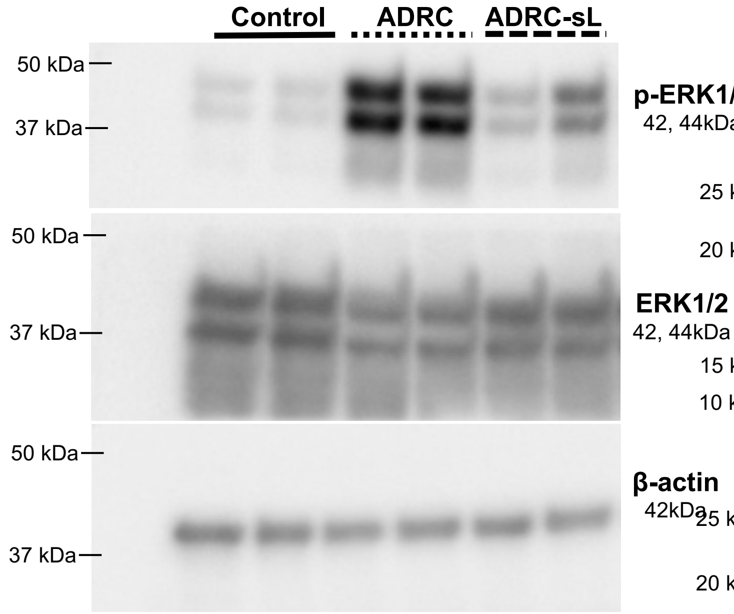

Figure 5I (set 3)

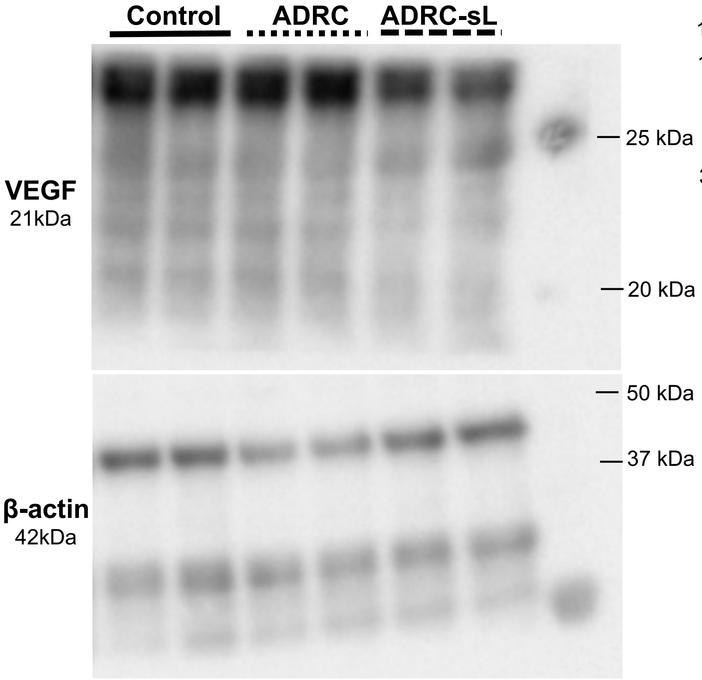

Figure 5L

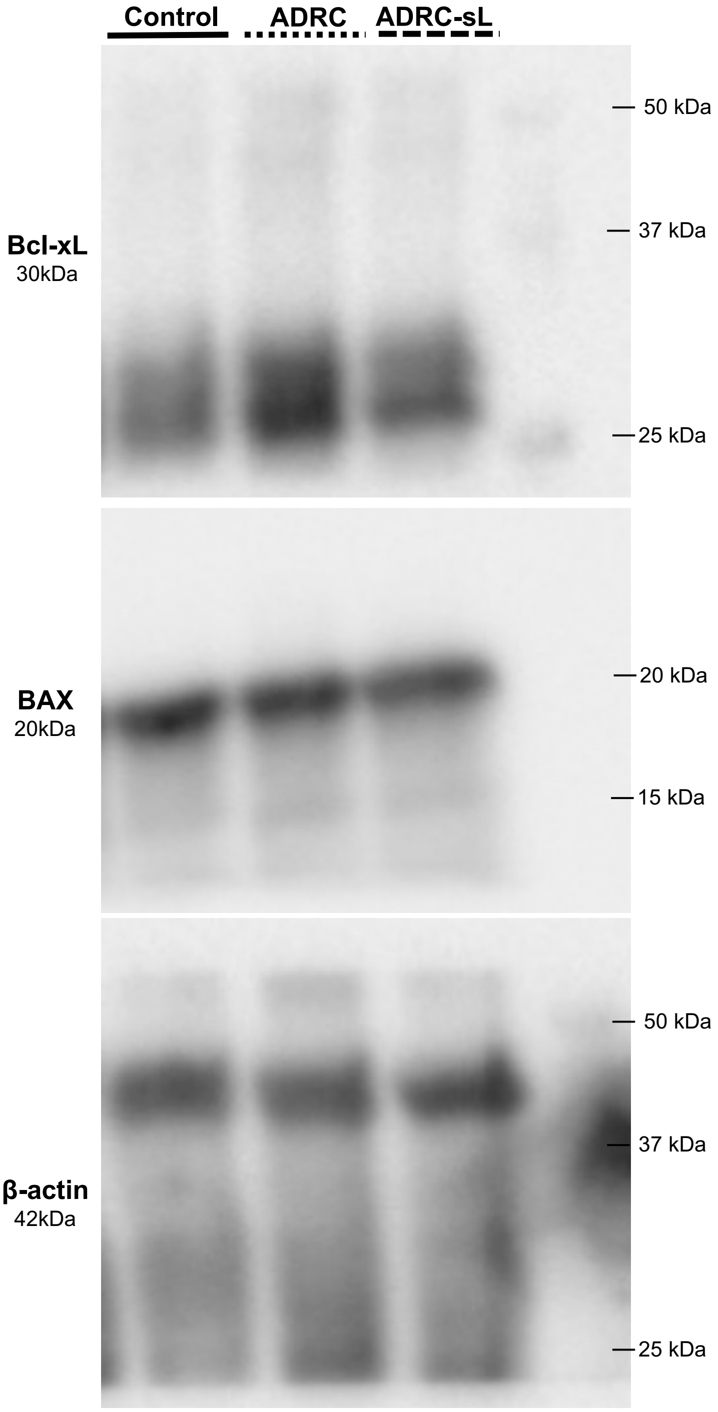
Control
ADRC
ADRC-sL
Bcl-xL
30kDa
50 kDa
37 kDa
25 kDa
BAX
20kDa
20 kDa
15 kDa
β-actin
42kDa
50 kDa
37 kDa
25 kDa

Figure 6I

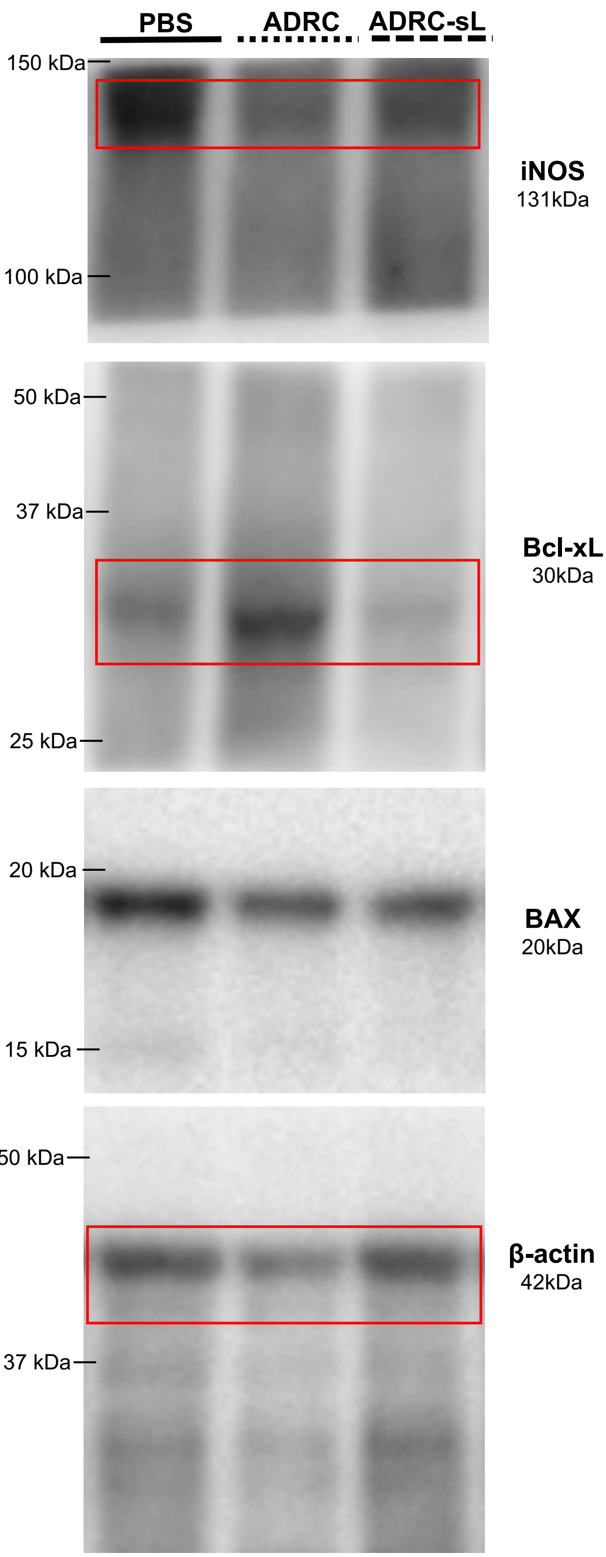

PBS
ADRC
ADRC-sL
150 kDa
100 kDa
iNOS
131kDa
50 kDa
37 kDa
25 kDa
Bcl-xL
30kDa
20 kDa
15 kDa
BAX
20kDa
50 kDa
37 kDa
β-actin
42kDa

Supplemental Figure 2B

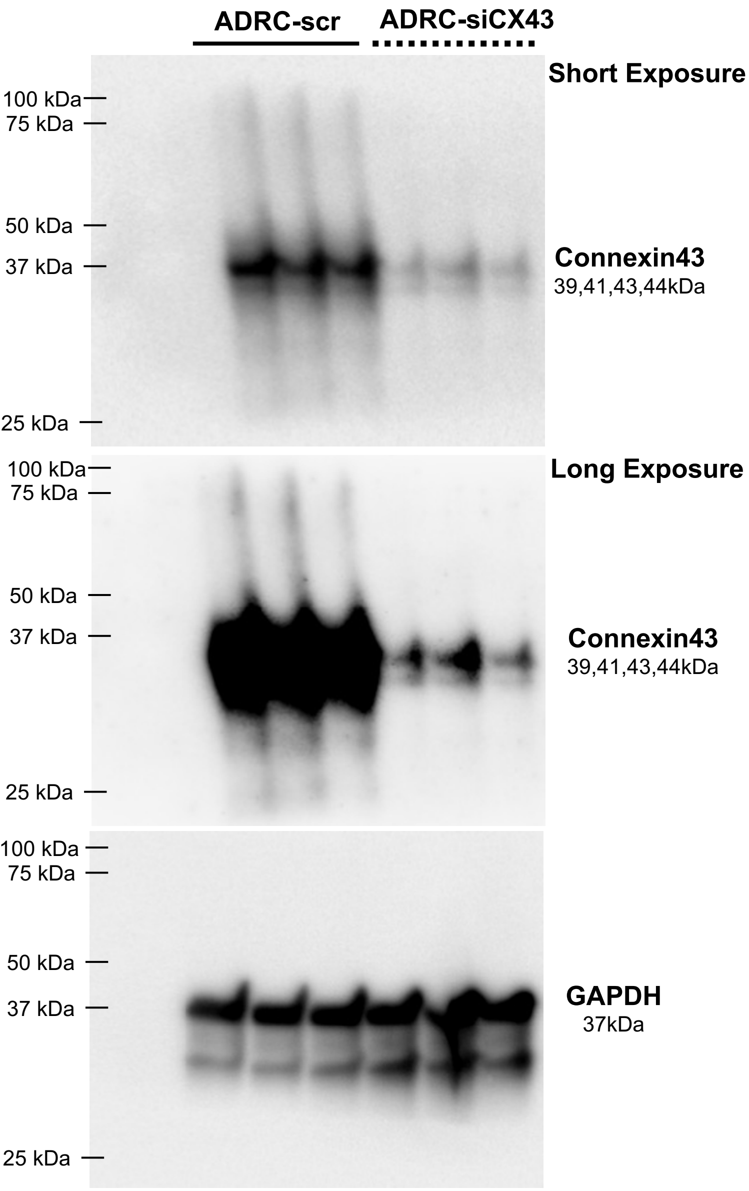

Supplemental Figure 2D

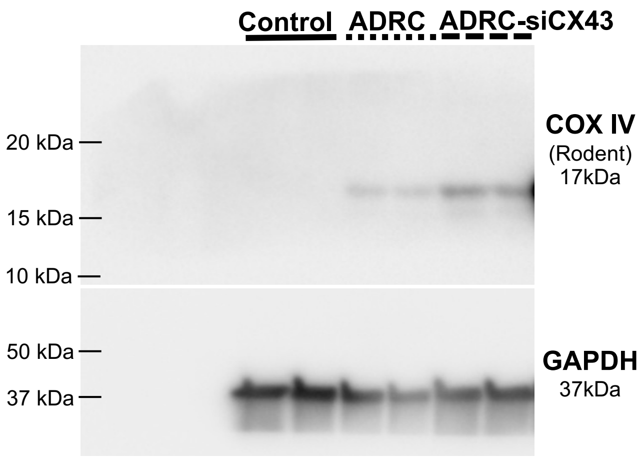

Supplemental Figure 2F

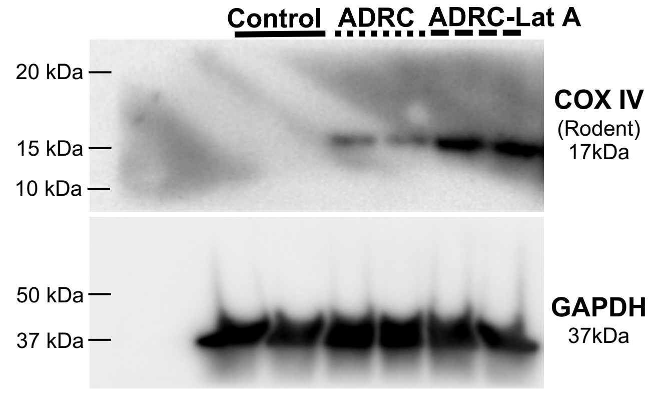

Supplemental Figure 4B

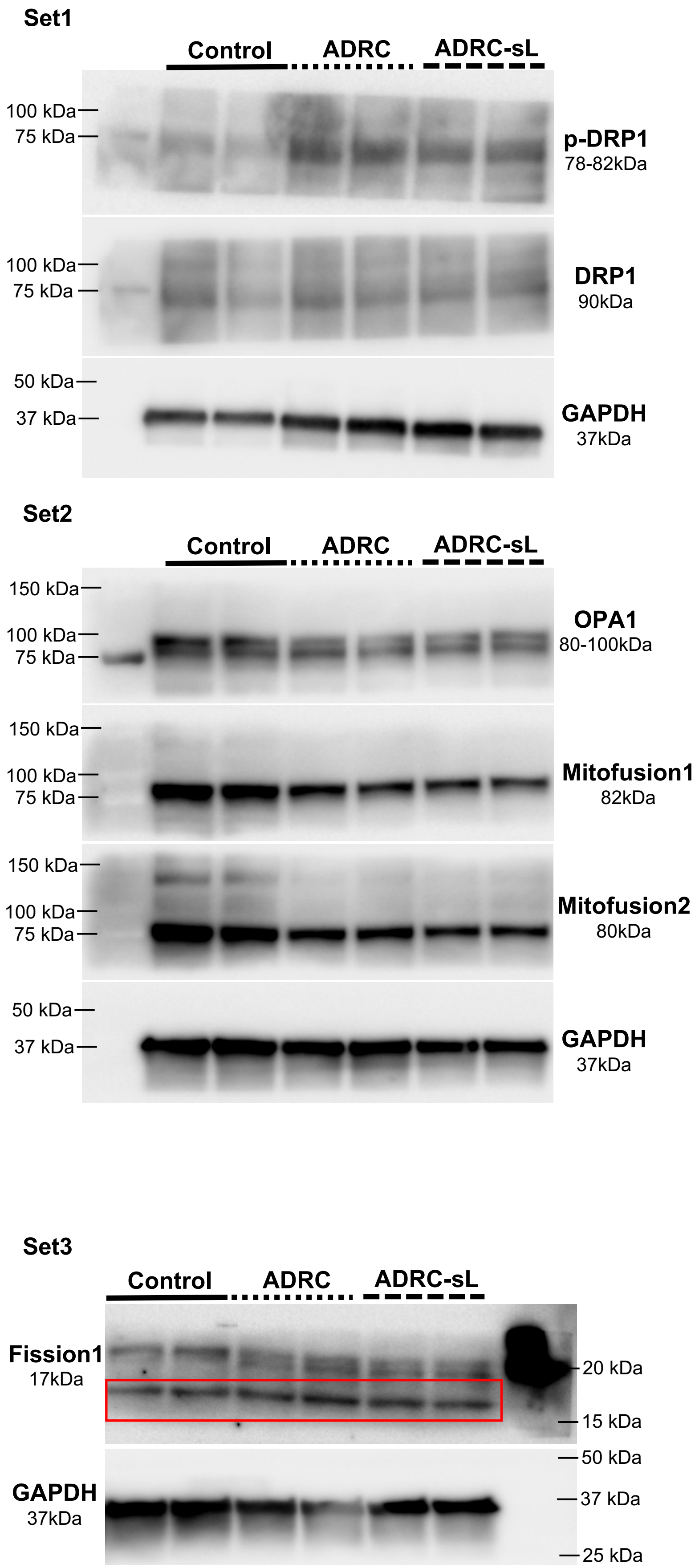

Supplement: Supplementary file 1 — Supplementary file1 (PDF 736 kb) [file 10456_2025_10001_MOESM1_ESM.pdf]
