# Supplementary material for: Mitochondrial transfer from adipose-derived regenerative cells contributes therapeutic angiogenesis in a murine hindlimb ischemia model
Source: Angiogenesis. 2025 Sep 10;28(4):49. doi: 10.1007/s10456-025-10001-z (PMC12423147; doi:10.1007/s10456-025-10001-z)
Supplement: Supplementary file 2 — Supplemental Fig. 1 Mitochondrial transfer from Mitochondria-GFP-labeled ADRC in a hindlimb ischemia mouse model. (A) Schematic illustration for the administration of Mitochondria-GFP-labeled ADRCs and mitochondrial transfer evaluation in a hindlimb ischemia mouse model. (B) Representative adductor muscle images stained by CD31 (red). The transferred mitochondria (green) originating from ADRCs are labeled with white arrows. Scale bar represents 100µm (20x magnification). (C) Representative adductor muscle images stained by CD68 (red). The transferred mitochondria (green) originating from ADRCs are labeled with white arrows. Scale bar represents 100µm (20x magnification); Supplemental Fig. 2 Different modes of mitochondrial transfer from ADRC to HUVEC. (A) Representative images of ADRCs coculturing with HUVECs stained by Connexin43 (green) and WGA (red) after 12H/24R. Scale bar represents 100µm (20x magnification). (B) Representative western blots and quantification of Connexin43 and GAPDH in ADRC-scr and ADRC-siCX43 (n=6 per group). (C) Quantification of mouse mtDNA expression in in PBS, ADRC and ADRC-siCX43 group after 12H/24R. Control indicates HUVEC only group, ADRC indicates sorted HUVECs from coculture of ADRCs and HUVECs group and ADRC-siCX43 indicates sorted HUVECs from coculture of ADRCs (with siCX43 transfection) and HUVECs group (n=5 per group). (D) Representative western blots of rodent-specific COX IV and GAPDH in PBS, ADRC and ADRC-siCX43 group after 12H/24R. (E) Quantification of mouse mtDNA expression in in PBS, ADRC and ADRC-Lat A group after 12H/24R. Control indicates HUVEC only group, ADRC indicates sorted HUVECs from coculture of ADRCs and HUVECs group and ADRC-siCX43 indicates sorted HUVECs from coculture of ADRCs (with Lat-A treatment) and HUVECs group (n=5 per group). (F) Representative western blots of rodent-specific COX IV and GAPDH in PBS, ADRC and ADRC-Lat A group after 12H/24R. (G) Representative electron microscopy images of mitochondria [file 10456_2025_10001_MOESM2_ESM.pdf]

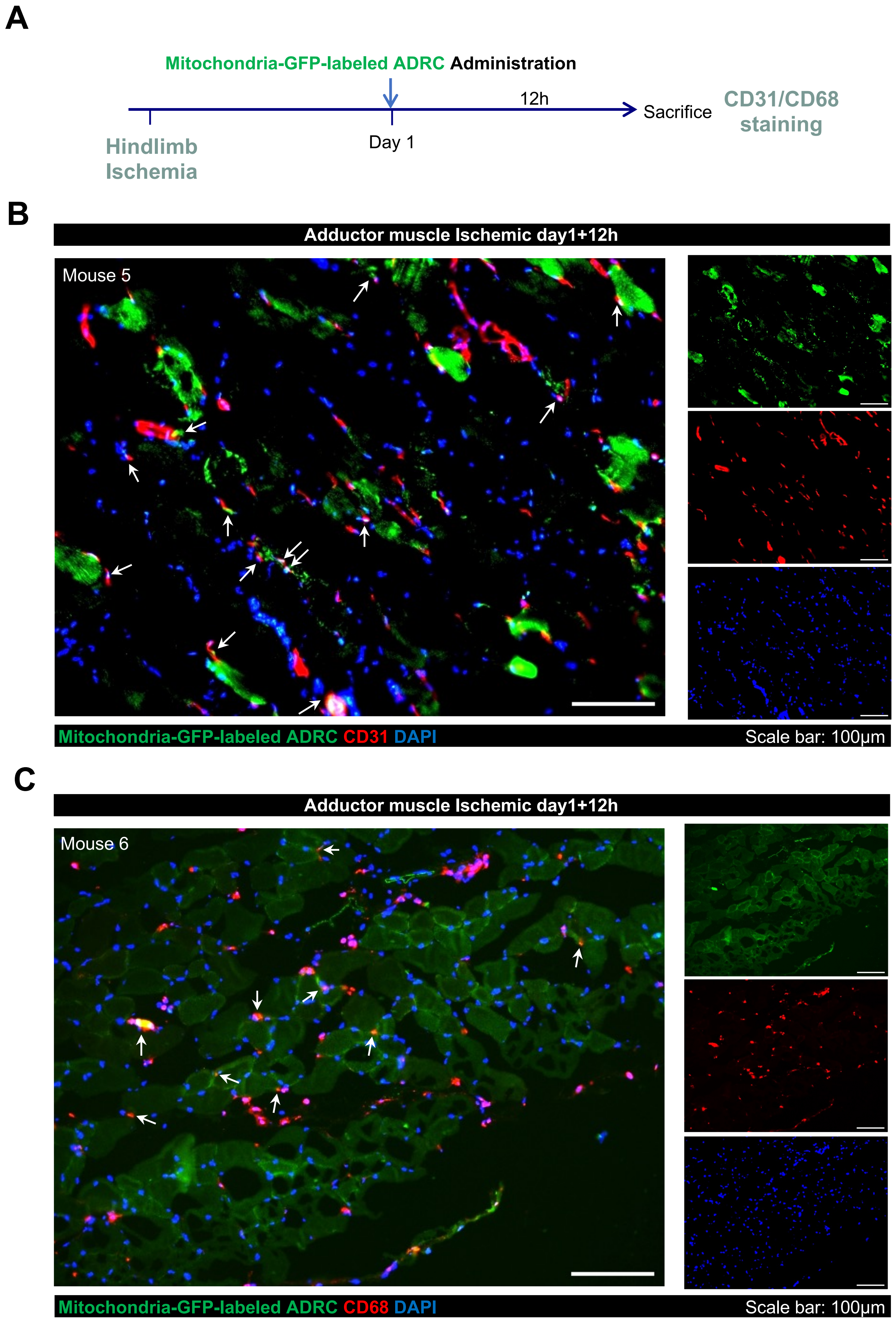

Supplemental Figure 1

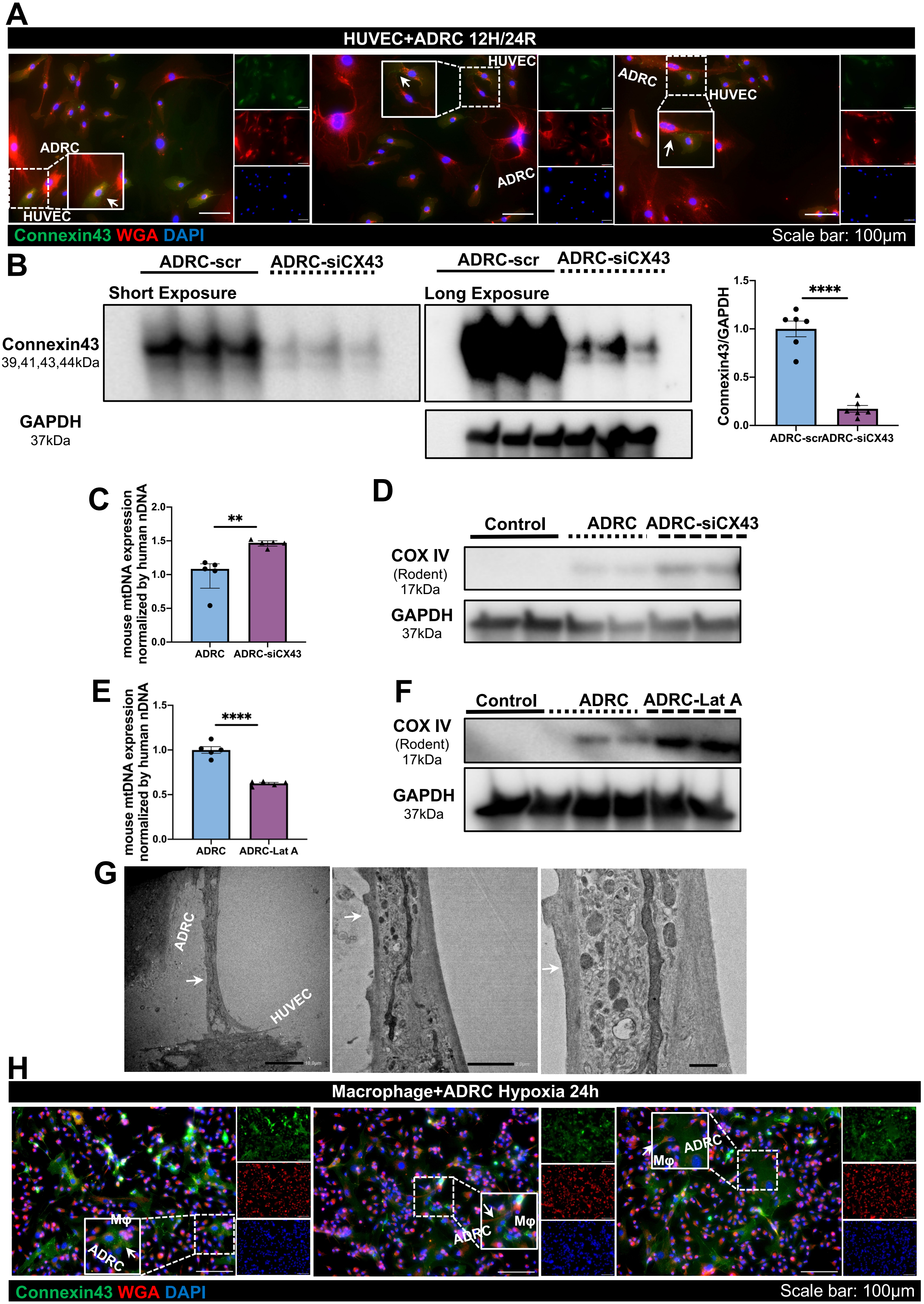

Supplemental Figure 2

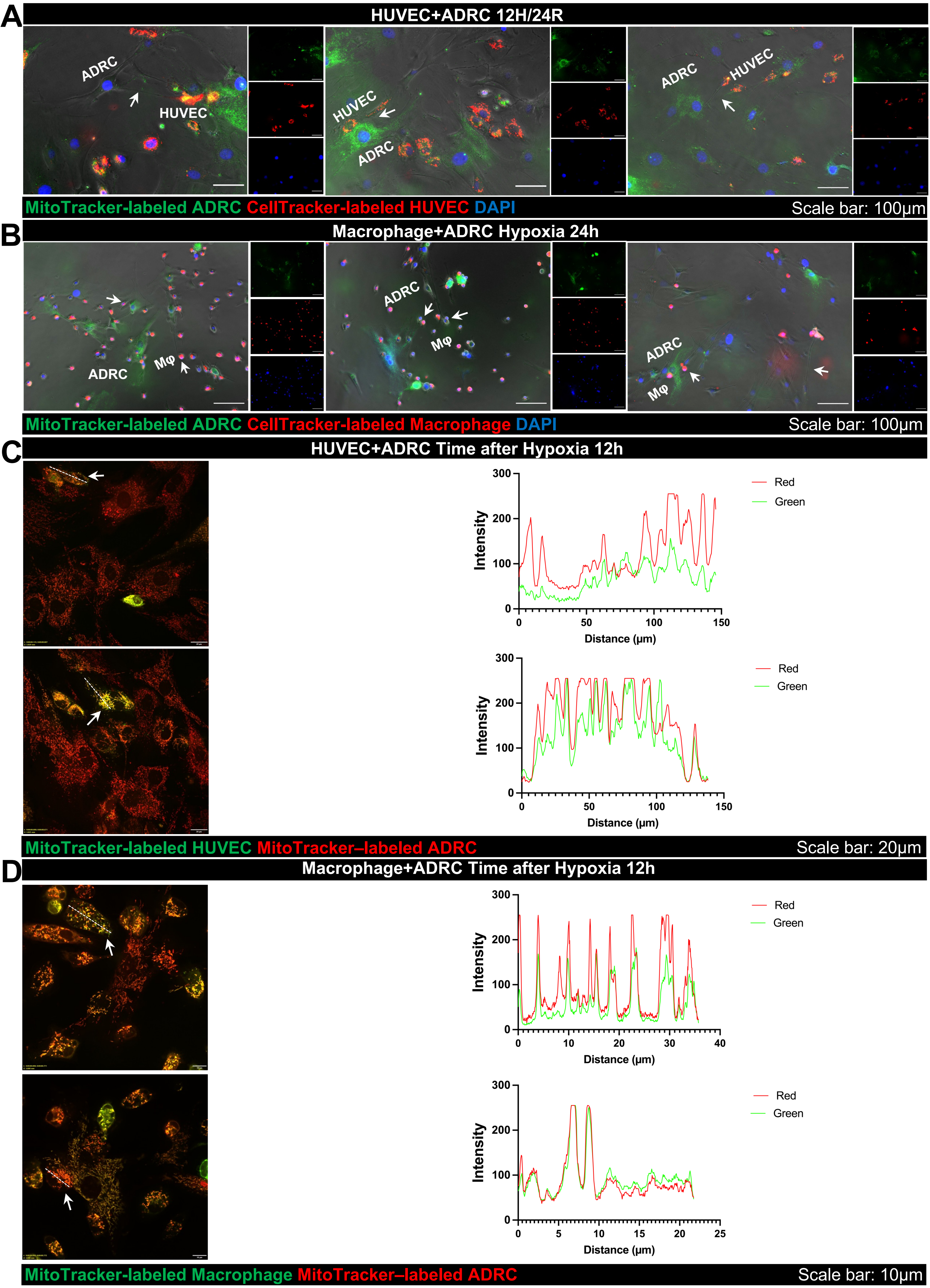

Supplemental Figure 3

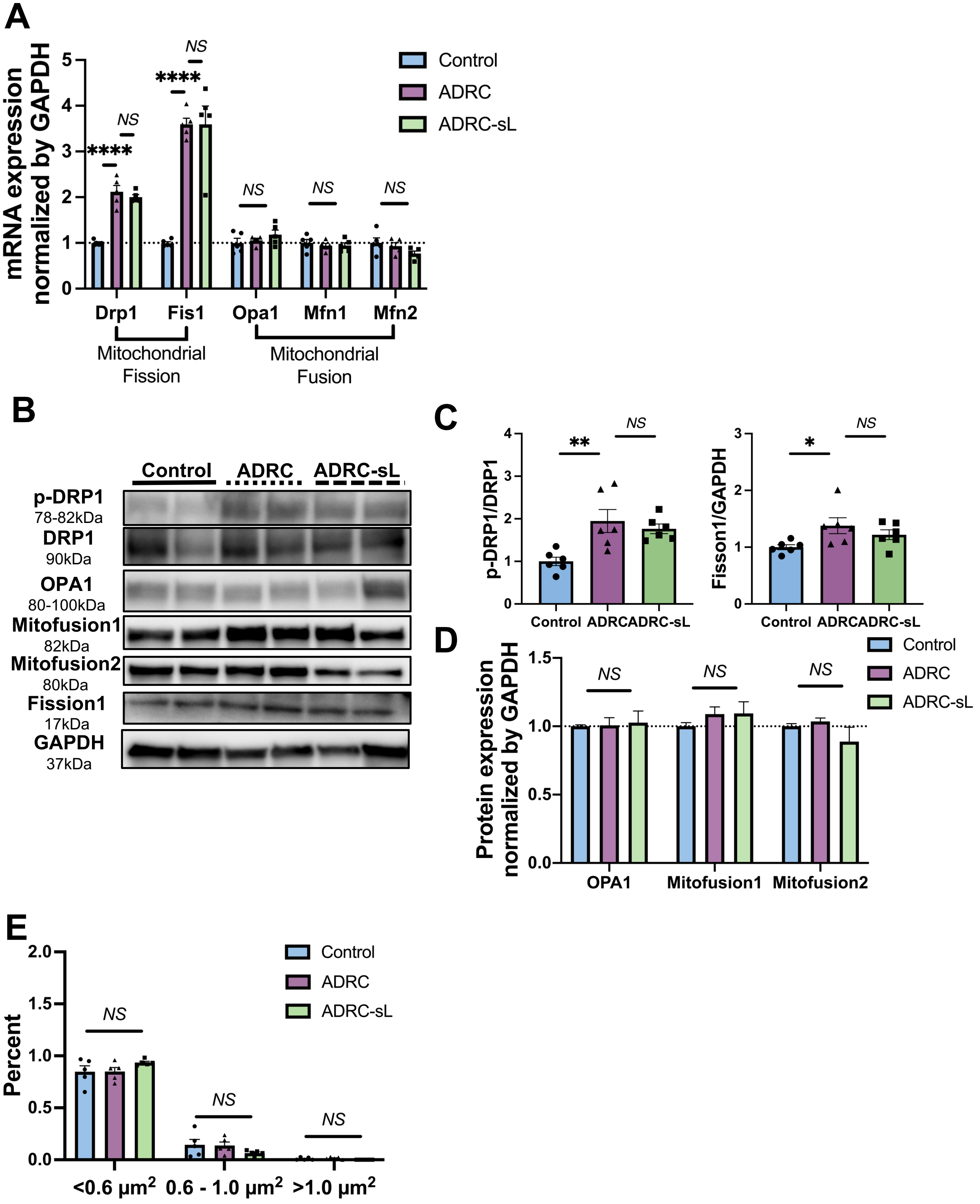

Supplemental Figure 4

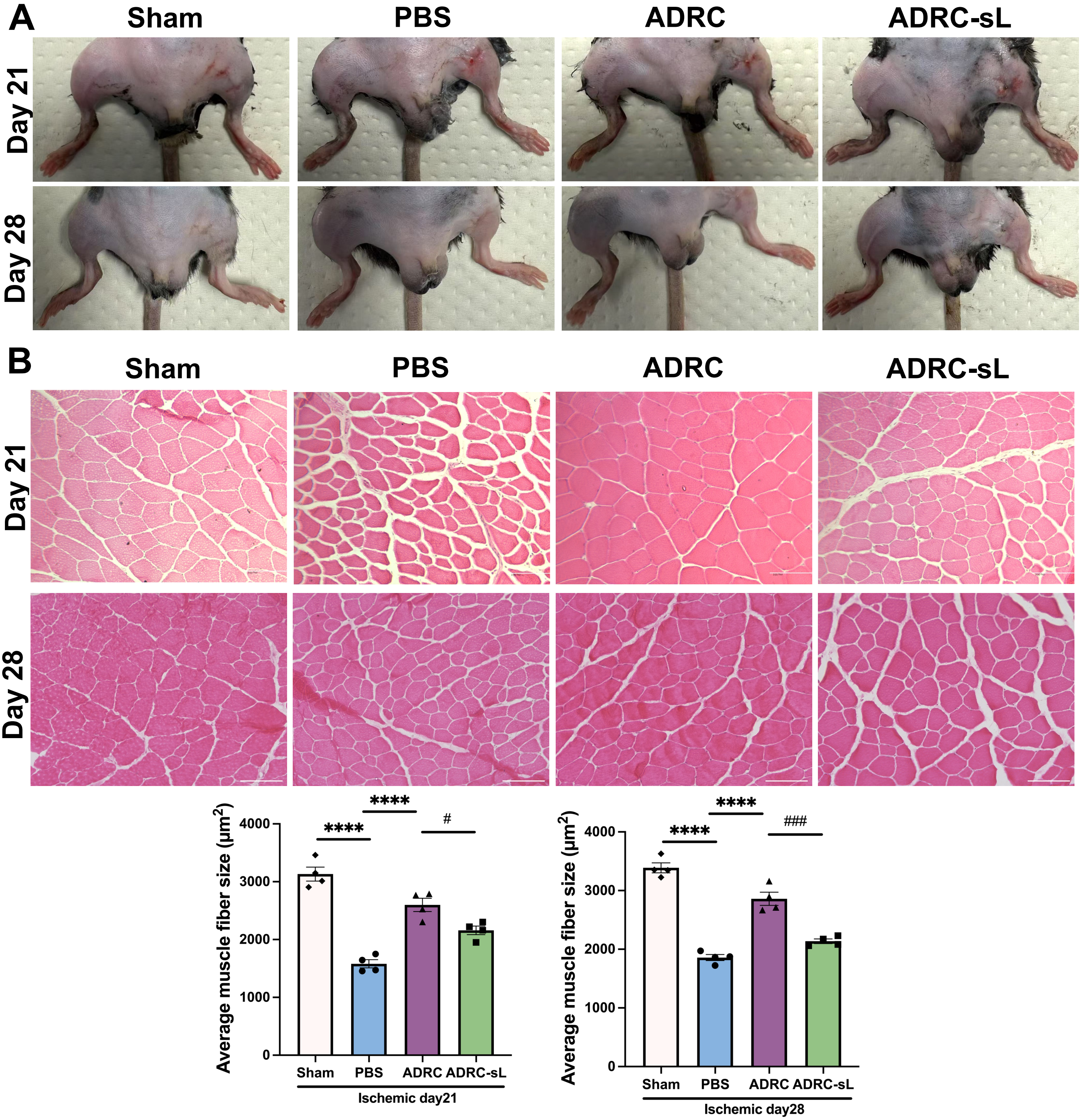

Supplemental Figure 5

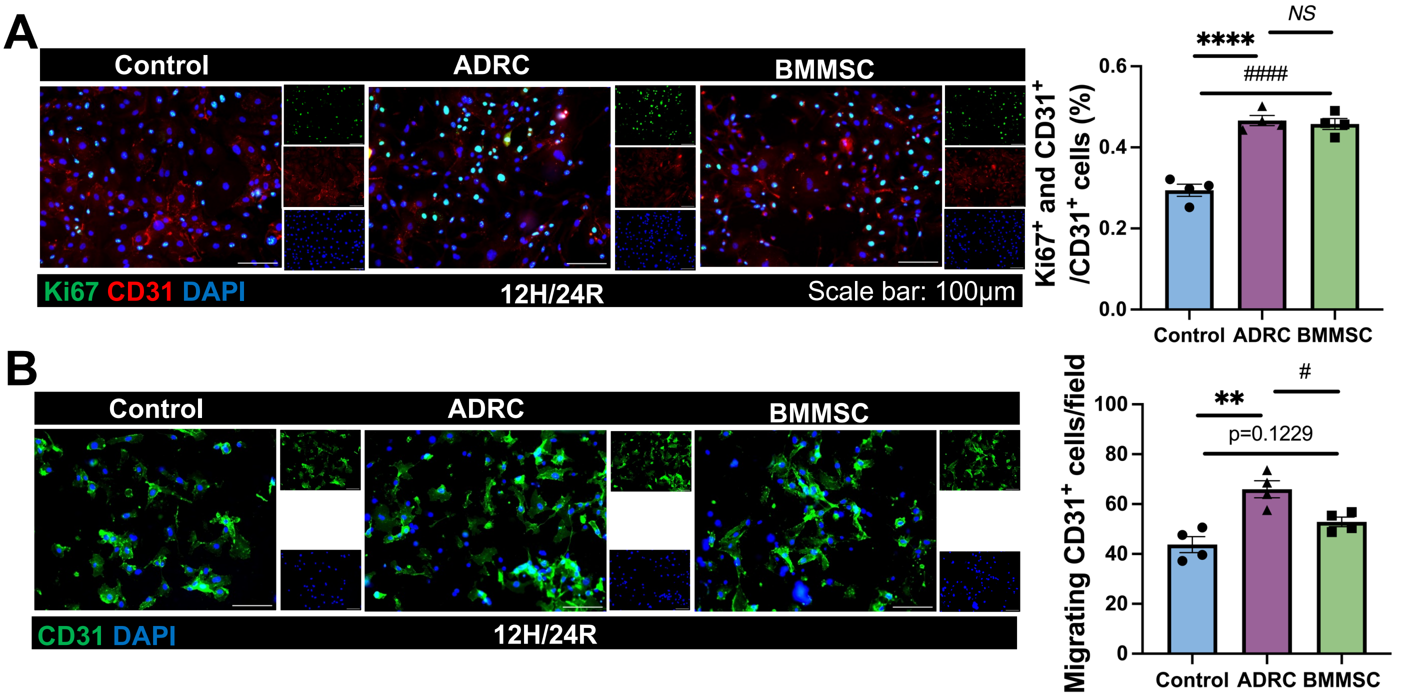

Supplemental Figure 6

A

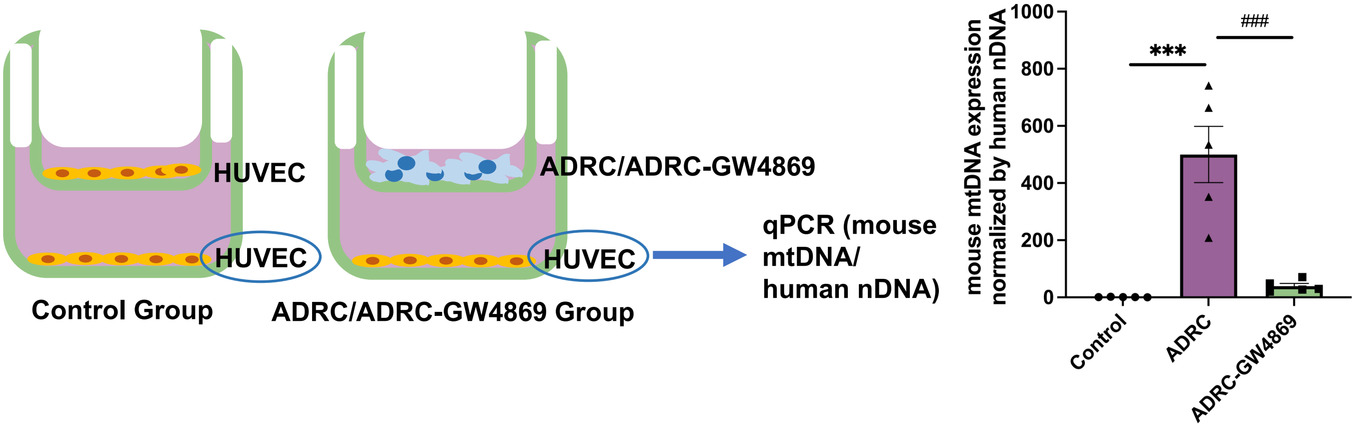

Supplemental Figure 7

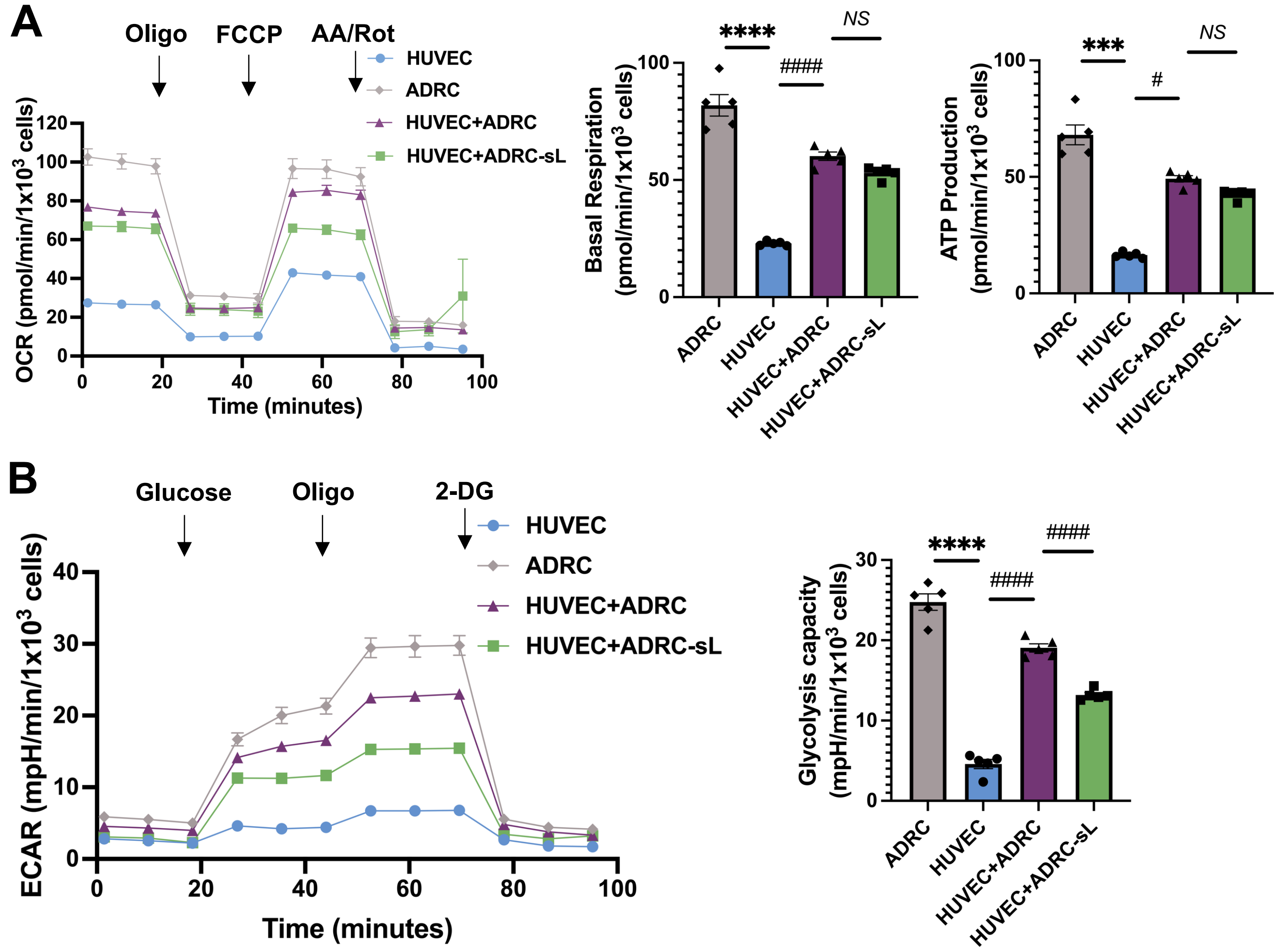

Supplemental Figure 8

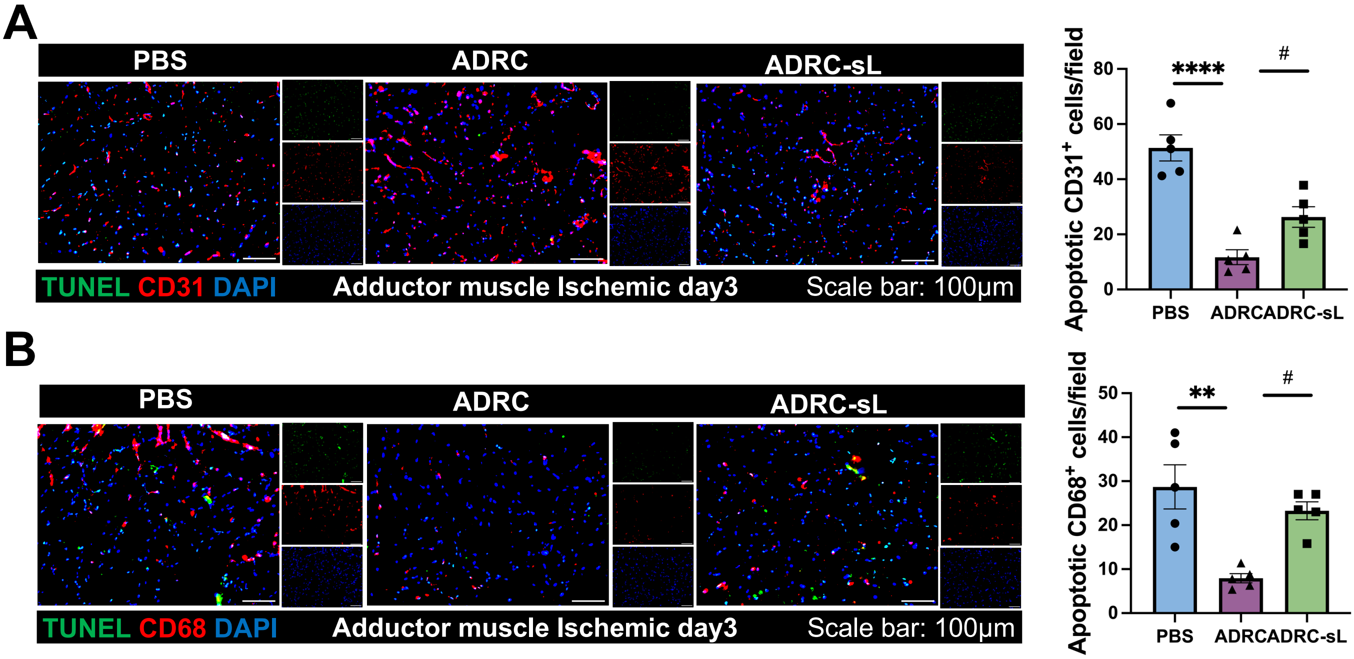

Supplemental Figure 9

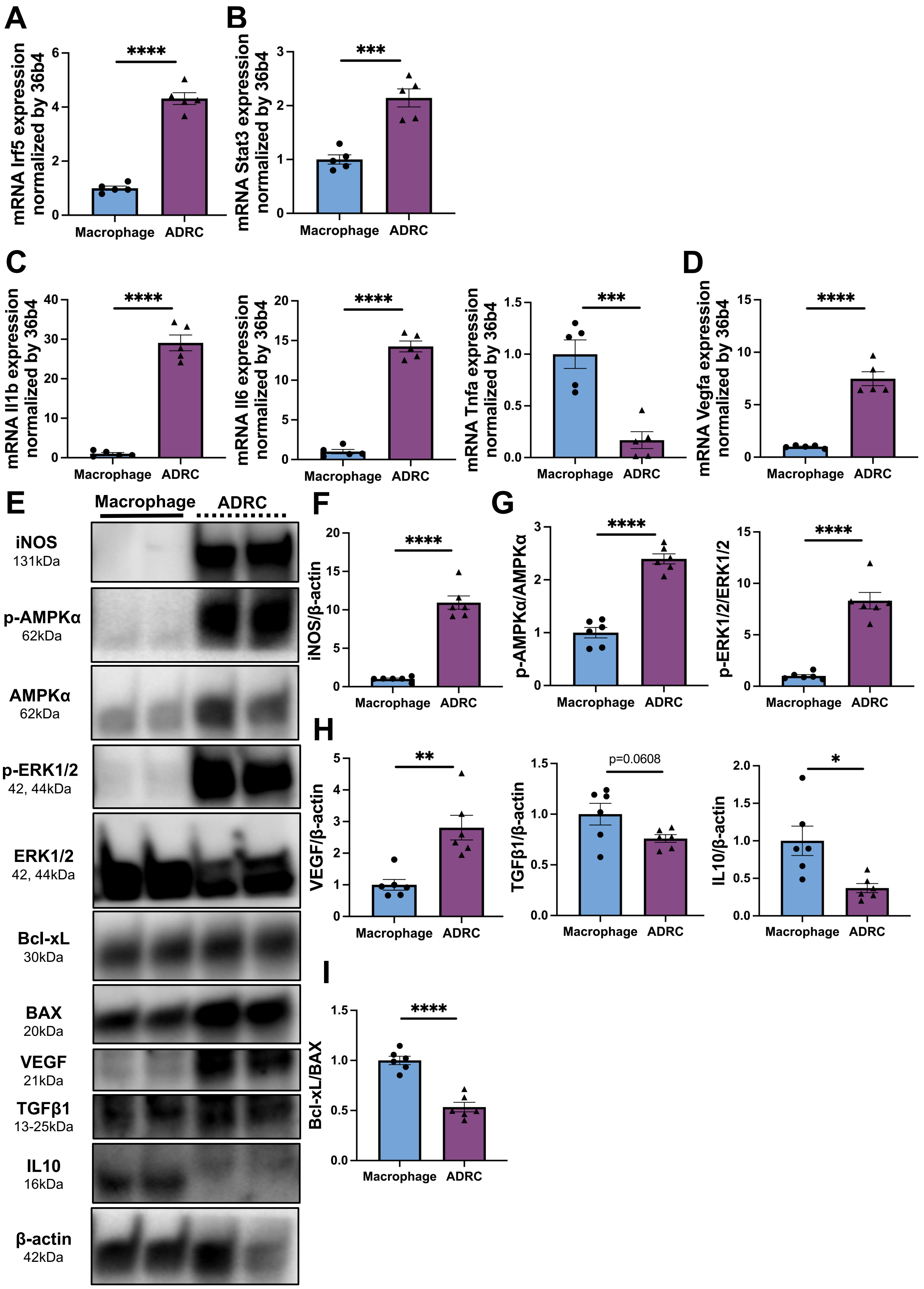

Supplemental Figure 10

Table S1. Human and mouse primers were used for qPCR.

Target	Forward primer	Reverse primer
Human		
Drp1	ACCCGGAGACCTCTCATTCT	TGACAACGTTGGGTGAAAAA
Fis1	GATGACATCCGTAAAGGCATCG	AGAAGACGTAATCCCGCTGTT
Gapdh	GTCTCCTCTGACTTCAACAGCG	ACCACCCTGTTGCTGTAGCCAA
Mfn1	GAGGTGCTATCTCGGAGACAC	GCCAATCCCACTAGGGAGAAC
Mfn2	CACATGGAGCGTTGTACCAG	TTGAGCACCTCCTTAGCAGAC
mtDNA	CCCCACAAACCCCATTACTAAACCCA	TTTCATCATGCGGAGATGTTGGATGG
nucDNA	CGAGTCGTCTTTCTCCTGATGAT	TTCTGGATTCCAATGCTTCGA
Opa1	TGTGAGGTCTGCCAGTCTTTA	TGTCCTTAATTGGGGTCGTTG
Pgc1a	CCAAAGGATGCGCTCTCGTTCA	CGGTGTCTGTAGTGGCTTGACT
Mouse		
Arg1	CATTGGCTTGCGAGACGTAGAC	GCTGAAGGTCTCTTCCATCACC
Cd11c	GCCAGGATGACCTTAGTGTCG	CAGAGTGACTGTGGTTCCGTAG
Cd163	GGCTAGACGAAGTCATCTGCAC	CTTCGTTGGTCAGCCTCAGAGA
Cd206	GTTCACCTGGAGTGATGGTTCTC	AGGACATGCCAGGGTCACCTTT
Gapdh	ACCCAGAAGACTGTGGATGG	CACATTGGGGGTAGGAACAC
Il1b	TGGACCTTCCAGGATGAGGACA	GTTCATCTCGGAGCCTGTAGTG
Il6	AGTTGCCTTCTTGGGACTG	TCCACGATTTCCCAGAGAAC
iNOS	GAGACAGGGAAGTCTGAAGCAC	CCAGCAGTAGTTGCTCCTCTTC
Irf5	CCTACAGAACCACTCTTGCCTG	CCTTGTGGGTTGCTGATGGTGA
Kif5b	GCGAGATGAAGTGGAGGCAAAG	CTCTTGGTCTGTAGCCTTCAGC
mtDNA	CCCAGCTACTACCATCATTCAAGT	GATGGTTTGGGAGATTGGTTGATGT
Rala	ACAGAGCTGACCAGTGGAACGT	GCTGTCTTCCATCTTTCTGGCTC
Rhot1	CTCCACCTCAAGCCTTCACTTG	GCTCTTGAGGTCAGCTTGTGTC
Stat3	AGAACCTCCAGGACGACTTTG	TCACAATGCTTCTCCGCATCT
Tgfb1	TGATACGCCTGAGTGGCTGTCT	CACAAGAGCAGTGAGCGCTGAA
Tnfa	TGATCGGTCCCCAAAGGGAT	TGTCTTTGAGATCCATGCCGT
Tnfaip2	TTCGCCTGCAAGATTCCAGTGC	GCTCCTGACTTCACTGCTTGGT
Vegfa	CAGGCTGCTGTAACGATGAA	AATGCTTTCTCCGCTCTGAA
Ym1	TACTCACTTCCACAGGAGCAGG	CTCCAGTGTAGCCATCCTTAGG
36b4	GCTTCGTGTTCACCAAGGAGGA	GTCCTAGACCAGTGTTCTGAGC

Table S2. Antibodies used for the experiments.

Target antigen	Vendor	Catalog number	Working concentration
AMPKa	Cell Signaling	2532S	1:1000
p-AMPKa	Cell Signaling	2531S	1:1000
BAX	Cell Signaling	14796S	1:1000
Bcl-xL	Cell Signaling	2764S	1:1000
β-Actin	Cell Signaling	4967S	1:1000
Connexin43	Cell Signaling	3512S	1:1000
Cox iv	Cell Signaling	38563S	1:1000
DRP1	Abcam	ab56788	1:2000
p-DRP1	Cell Signaling	3455S	1:1000
ERK1/2	Cell Signaling	4695S	1:1000
p-ERK1/2	Cell Signaling	4370S	1:1000
Fission1	Merck	3271978	1:1000
GAPDH	Cell Signaling	2118S	1:2000
iNOS	Abcam	Ab178945	1:1000
IL10	R & D	AF519	1:1000
Mitofusion1	Cell Signaling	14739S	1:1000
Mitofusion2	Cell Signaling	11925S	1:1000
OPA1	Cell Signaling	67589S	1:1000
PGC1a	Cell Signaling	2178S	1:1000
TFAM	Cell Signaling	7495S	1:1000
TGFb1	Santa Cruz	sc-130348	1:1000
VEGF	Santa Cruz	sc-7269	1:1000
Polyclonal Goat Anti-Mouse Immunoglobulins HRP	Agilent technologies	P0447	1:5000
Polyclonal Goat Anti-Rabbit Immunoglobulins HRP	Agilent technologies	P0448	1:5000
Polyclonal Rabbit Anti-Goat Immunoglobulins HRP	Agilent technologies	P0449	1:5000

Table S3. Raw data for Figure S4D

	OPA1				Mitofusion1				Mitofusion2	
Control	ADRC	ADRC-sL		Control	ADRC	ADRC-sL		Control	ADRC	ADRC-sL
1.03105	0.94624	0.93298		1.03981	0.88157	0.79272		0.97708	1.07672	1.11781
0.96895	0.78897	1.02970		0.96019	1.11109	1.25066		1.02292	0.99343	1.11124
1.02217	0.95389	0.88085		1.02321	1.09118	1.26897		0.94813	1.10432	1.00295
0.97783	1.10616	1.44163		0.97679	1.25748	1.27229		1.05187	1.07862	0.95803
1.02085	1.16909	0.90653		1.09566	1.03330	0.88853		1.04664	1.00732	0.52584
0.97915	1.07434	0.96674		0.90434	1.16506	1.09110		0.95336	0.95694	0.61437
